# Species‐environment relationships of coastal diatoms from the Sept‐Îles region, Gulf of St‐Lawrence (Québec, Canada)

**DOI:** 10.1111/jpy.70094

**Published:** 2025-09-29

**Authors:** Emilie Arseneault, Émilie Saulnier‐Talbot

**Affiliations:** ^1^ Biology Department, Québec‐Océan and Institute of Integrative Biology and Systems Université Laval Quebec City Quebec Canada; ^2^ Geography Department Université Laval Quebec City Quebec Canada

**Keywords:** benthic, bioindicators, ecology, temperature, transfer function

## Abstract

As climate change is having increasingly visible impacts on coastal regions, it is urgent to better understand its effects on the state of ecosystems and the services they provide. To assess the direction and magnitude of change in the high‐use waters of the Sept‐Îles region in the Gulf of St‐Lawrence, we sampled 35 sites along the coast and explored the relationship between surface diatom assemblages and 21 environmental variables. Temperature (14.62%), Secchi depth (12.97%), dissolved silica (6.91%), and magnesium (6.23%) were determined to be significant and independent variables explaining variation in diatom distribution, together explaining 28.6% of the assemblage variance. Temperature and Secchi depth met the λ_1_/λ_2_ > 0.5 criterion, but only temperature was selected to develop a transfer function, as these two variables were strongly correlated. A diatom‐based temperature transfer function was then developed using weighted‐averaging partial least squares (WAPLS; 3; *r* ^2^ = 0.59, RMSEP = 0.45°C), based on a training set of 32 sites and 55 benthic taxa. However, the model exhibited sensitivity to spatial autocorrelation and may have been influenced by secondary and unmeasured variables. Despite these limitations, the model provides valuable insight into the spatial variability of diatom assemblages and offers potential for paleoenvironmental reconstructions. For optimal inferences, the model should be applied within the studied area. This study contributes to understanding how coastal diatom assemblages respond to environmental gradients and highlights the importance of diatom‐based monitoring to assess ongoing ecological changes in aquatic environments.

**Abbreviations**
BSI: Bay of Sept‐Îles
CTD: conductivity‐temperature‐depth
DCA: detrended correspondence analysis
DIC: differential interference contrast
DIP: dissolved inorganic phosphorus
DSi: dissolved silica
PAR: photosynthetically active radiation
PCA: principal component analysis
RDA: redundancy analysis
RMSEP: root mean square error of prediction
RNE: random, neighbor, environment deletion analysis
TDN: total dissolved nitrogen
TDP: total dissolved phosphorus
TDS: total dissolved solids
TP: total phosphorus
WA: weighted average
WAPLS: weighted‐averaging partial least squares
W‐E: West–East

## INTRODUCTION

Estuaries and coastal environments are some of the most densely populated areas in the world and have been impacted by human activities for centuries. They face pressures from rapid climate change and anthropogenic factors, including rising water temperatures and sea level, habitat loss, eutrophication, acidification, and probable increasing strength and frequency of storms (He & Silliman, 2019; Lotze et al., 2006). In these mutating coastal ecosystems, alterations in species communities often occur, and those changes can serve as reliable indicators of the effects of environmental changes on ecosystem structure and function (Desrosiers et al., 2013).

The short life cycle of diatoms, a group of microscopic siliceous algae, enables them to respond rapidly to disturbances in their environments, resulting in changes in assemblage. Diatoms are also sensitive to changes in various environmental variables. These features, together with the widespread presence of diatoms in aquatic ecosystems around the world, make diatoms powerful bio‐indicators (Birks, 2010; Julius & Theriot, 2010). They are regularly used for various types of studies, such as to assess the state of the environment or to trace paleo‐environmental conditions through qualitative and quantitative inferences, mostly in freshwaters (Battarbee et al., 2008; Birks et al., 1990; Duda et al., 2023). Because of their siliceous cell walls, diatoms can be preserved in sediments through time, making paleo‐environmental studies possible (Julius & Theriot, 2010). Diatoms are commonly used to develop calibration models that define modern species–environment relationships. These models, also known as transfer functions, identify the most significant environmental parameters shaping diatom assemblages (Birks, 2010). Through this approach, optima and tolerances are estimated for each taxon, enabling the quantitative inferences of present and past environmental conditions. As such, diatom‐based models, despite their shortcomings (Juggins, 2013), can be powerful tools for tracking ecosystem responses to change (Leventer et al., 2012). This approach has been largely applied to lakes (e.g., Alibert et al., 2025; Fallu et al., 2000; Reavie et al., 2006; Tremblay et al., 2014; Weckström et al., 1997), less so to coastal and marine environments (Wachnicka et al., 2010; Weckström et al., 2004). However, the development and application of transfer functions are challenging and have repeatedly been called into question (Anderson, 2000; Juggins, 2013; Telford & Birks, 2009). They remain, nonetheless, helpful in many cases, especially to explore the links between biotic and abiotic components of ecosystems.

The St. Lawrence Seaway System is a densely populated region in Eastern Canada. Most of the population of Québec and of the Atlantic provinces inhabit the shores of the Gulf and St. Lawrence Estuary (Schloss et al., 2017). This region provides access to several commercial ports and is exposed to many types of urban, maritime, and industrial activities. Consequently, the environment is exposed to anthropogenic disturbance (Beauchesne et al., 2020; Dreujou et al., 2020). The Bay of Sept‐Îles (BSI), located in the northern region of the Gulf, houses the most important mineral port in North America and is subject to several potential sources of contamination because of industrial activities and marine traffic (Carrière & Le Hénaff, 2018). Since 2013, the state of the BSI's environment has been monitored in order to determine how it is affected by urban, port, industrial, and maritime activities (Ferrario et al., 2022). At present, the BSI is also part of a program allowing near real‐time monitoring (Carrière & Dreujou, 2024). The use of bioindicators in the region is currently limited, but an effort to develop this approach is ongoing (e.g., Arseneault et al., 2023; Joshi et al., 2025).

Obtaining autecological information about coastal diatoms in a dynamic environment like Sept‐Îles and knowing which environmental gradient is influencing their distribution is essential in order to develop biological tools for environmental assessment. The scarcity of studies on coastal diatoms as indicators for environmental biomonitoring currently limits their use. It is therefore important to acquire more knowledge about the autecological preferences and distribution of diatoms in high‐use coastal areas, to improve science‐based monitoring and management. This study aimed to develop diatom‐based inference models as valuable biomonitoring tools to inform the science‐based management of the Bay of Sept‐Îles and its surroundings in the context of current global changes.

The main objectives of this study were (1) to explore the relationships between modern diatom species and the associated aquatic parameters and (2) to determine which variables were significantly and independently shaping the assemblages and distribution, to develop diatom‐based calibration models.

## MATERIALS AND METHODS

### Study area

**FIGURE 1:**
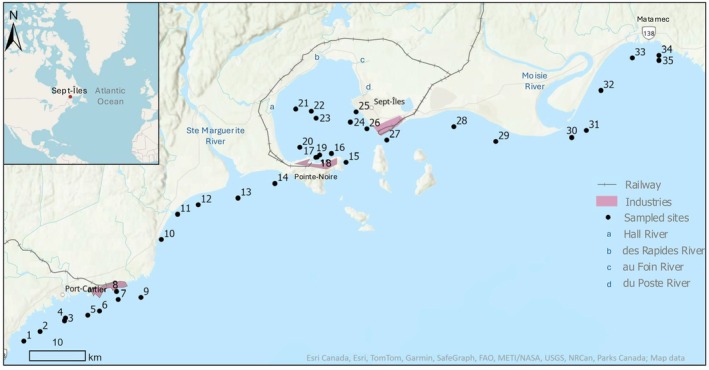
Map of the Sept‐Îles area, showing the 35 sampling sites and the elements mentioned in the text.

The study area was a transect of 80 km along the coastline of the Sept‐Îles region, from Port‐Cartier to Matamec (Figure 1). Located in the northern Gulf of St. Lawrence and characterized by a subarctic climate (Demers et al., 2018), the region is highly dynamic. Usually ice‐covered from November to April (Demers et al., 2018), the area now experiences earlier melt and unpredictable ice conditions due to climate breakdown (Allard, 2024). The region lies within an upwelling zone (Bourque & Kelley, 1995; Doyon & Ingram, 2000), with typical phytoplankton blooms in spring and autumn (Lefebvre, 2023) and a possible deep chlorophyll *a* maximum in summer (E. Arseneault, N. Joshi, J. Carrière, É. Saulnier‐Talbot, unpublished data). The area exhibits an estuarine circulation pattern with surface currents (2–10 m) flowing seaward at an average speed of 17.4 cm · s^−1^ and shoreward in deeper waters (10–30 m). The estimated bulk flushing time of the bay ranges from 2 to 9 days (Shaw et al., 2022). The region is under a semi‐diurnal tidal system, with tidal amplitudes ranging from 0.39 to 3.6 m in Port‐Cartier and 0.43 to 3.36 in Sept‐Îles (Fisheries and Ocean Canada, 2025). Freshwater inputs from various rivers and streams further shape the hydrography. Major contributors include the Ste‐Marguerite and Moisie rivers; within the BSI, rivers Hall, des Rapides, aux Foins, and du Poste contribute to an annual freshwater discharge of around 22 m^3^ · s^−1^ (Shaw et al., 2019). Diverse types of land use along the transect have been recorded, including industrial zones in Port‐Cartier and in the BSI, natural coastlines, and less developed areas in Matamec that feature an ecological reserve.

### Chemical and physical measurements

Study sites were selected along the coast following, more or less, the 20‐m bathymetric line. Each site was visited twice: between July 11, 2021 and July 13, 2021, and between July 19, 2022 and July 21, 2022. Conductivity, salinity, total dissolved solids (TDS), turbidity, pH, and temperature were measured in situ using a Hanna HI 9829 portable multiparameter meter. Water transparency was measured using a Secchi disk but only in 2021. Depth was measured using a sensor on the boat. Samples for chemical analysis were collected using bottles and were filtered using syringes and 45‐μm pore size filters on the day of collection, for analysis of dissolved metals (calcium, Ca; iron, Fe; magnesium, Mg; manganese, Mn; potassium, K; and sodium, Na; total dissolved phosphorus, TDP; total dissolved nitrogen, TDN; and dissolved silica, DSi). Chemical analyses were carried out by AGAT Laboratories in Quebec City, which is accredited by the Québec government and follows the ISO/IEC 17025:2017 standard (AGAT Laboratories, 2023). Samples for analysis of chloride (Cl), total phosphorus (TP), sulfate (SO_4_), ammonium (NH_4_), nitrate (NO_3_), and nitrite (NO_2_) were not filtered and were also analyzed by AGAT Laboratories. Analysis for dissolved inorganic phosphorus (DIP) was subcontracted to Bureau Veritas. The surface water parameters obtained from water column profiles of temperature, salinity, turbidity, chlorophyll *a* (Chl *a*), and photosynthetically active radiation (PAR) taken between May and October 2022 with a conductivity–temperature–depth (CTD) probe were also used in this study. Environmental characteristics of the study sites are given in Table 1.

**TABLE 1:** Environmental characteristics of the study sites.

	Mean	Median	Min	Max
Temperature (°C)	11.6	11.6	9.9	13
Chl *a* (mg · m^−3^)	0.9	0.9	0.6	1.3
Turbidity (FTU)	1.4	1.2	0.5	2.8
PAR (μmol photons · m^−2^ · s^−1^)	944.8	944.4	557.5	1393.4
Salinity	27.6	28	22.5	29.3
Depth (m)	20.3	19.6	4.3	53.5
pH	8.5	8.4	8.1	8.9
Conductivity (μS · cm^−1^)	44.1	44.9	34.4	46.9
TDS (mg · L^−1^)	22.1	22.5	15.9	23.5
Secchi (m)	10.8	11.2	4.2	18.4
Cl (g · L^−1^)	16.9	17.8	3.9	20
TP (μg · L^−1^)	74.4	70	25	235
SO_4_ (mg · L^−1^)	2309.2	2460	735.5	2815
NH_4_^+^ (μg · L^−1^)	178	190	50	245
Ca (mg · L^−1^)	296.3	293	84.1	365.5
Fe (μg · L^−1^)	50.1	20	20	519
Mg (mg · L^−1^)	951.3	960.5	259.3	1205
Mn (μg · L^−1^)	4.9	4	2	16
K (mg · L^−1^)	310.9	310.5	87.2	396
Na (g · L^−1^)	7.6	7.6	2.1	10.1
DSi (mg · L^−1^)	0.3	0.1	0	1.5

### Sampling and processing of sediment and diatoms

Surface sediment samples were collected in July 2021 from the same locations as the water parameter measurements using a modified ponar grab, which was designed to be opened from the top to allow surface sampling, preventing sediment mixing so as to maintain the stratigraphy. The surface sediments were scooped from within the grab and put into sample bags. They were kept refrigerated and in the dark until further analysis.

Sediment samples were treated with hydrochloric acid (37% HCl) to remove carbonates and with hydrogen peroxide (30% H_2_O_2_) to eliminate organic matter. After a 24‐h resting period, samples were heated to 80°C in a heating bath for approximately 6 h and cooled down to room temperature overnight. Samples were then rinsed with distilled water and decanted five times, with 24‐h intervals between each decantation, allowing the material to settle and facilitating the removal of the supernatant. Coverslips were prepared from 0.5 mL of the prepared solution and mounted using Meltmount© thermoplastic resin (Nevrova & Petrov, 2019), which has a refractive index of 1.74, similar to Naphrax. A minimum of 400 valves per sample were enumerated using a Leica DMRX light microscope equipped with differential interference contrast (DIC) illumination, under oil immersion. Broken diatom valves that had the middle and one extremity present were counted as one valve. All samples were processed as described in Arseneault et al. (2023), ensuring uniformity in microscope slide preparation. Diatom enumeration procedures and the same literature were used for identification, mainly sources related to coastal and marine environments (Campeau et al., 1999; Cardinal et al., 1984; Lee et al., 2015; Park et al., 2014; Pienitz et al., 2003; Poulin et al., 1984, 1990; Snoeijs, 1993; Snoeijs & Balashova, 1998; Snoeijs & Kasperovičienė, 1996; Snoeijs & Potapova, 1995; Snoeijs & Vilbaste, 1994; Witkowski et al., 2000).

### Data analysis

A total of 19 environmental variables was used for statistical analysis: Ca, Fe, Mg, Mn, K, Na, DSi, Cl, TP, SO_4_, NH_4_^+^, conductivity, salinity, TDS, pH, temperature, water transparency (Secchi disk), turbidity, depth, Chl *a*, and PAR. NO_3_ + NO_2_ was excluded from the statistical analysis, as concentrations were mostly below the detection limit (0.2 mg · L^−1^). Total dissolved nitrogen, TDP, and DIP were also excluded for the same reason. Specifically, TDN concentrations were below the detection limit (1000 μg · L^−1^) at all sites except sites 18 (2600 μg · L^−1^) and 19 (3100 μg · L^−1^). Over 80% of sites were below the detection limit for TDP (20 μg · L^−1^), and over 70% for DIP (30 μg · L^−1^). For statistical analyses, the following data were used: the mean values (between July 2021 and 2022) of Ca, Fe, Mg, Mn, K, Na, DSi, Cl, TP, SO_4_, NH_4_^+^, TDS; mean temperature and salinity from sampling in July 2021 and from May to October 2022; mean values of Chl *a* and PAR from May to October 2022; and pH and water transparency data from July 2021 only, due to technical issues encountered in July 2022.

Rare diatom species were excluded from statistical analysis. Only taxa with at least 1% of relative abundance and occurring in two or more samples were retained. Using the rioja package (Juggins, 2023), a diagram showing the 15 most frequent species throughout the transect was created, and two clustering analyses were performed using the factoextra package (Kassamara & Mundt, 2020): one of the environmental variables and sites and the other of the diatom assemblages and sites.

All analyses were performed using the R statistical software (R Core Team, 2022), and every ordination analysis used the vegan package (Oksanen et al., 2022). Each environmental variable was tested for skewness and log_10_(*x* + 1) transformed, if necessary, and scaled for ordinations. An exploratory principal component analysis (PCA) with every environmental variable was performed, offering an easy visualization of relationships between the sites and the different parameters. A Pearson correlation matrix was created using the corr_coeff function from the metan package (Olivoto & Lúcio, 2020) to identify significantly correlated (*r* > 0.8 with *p* < 0.001) and thus redundant variables (Fallu, 1998; Weckström & Juggins, 2005). Based on the results, groups of correlated variables were formed, to retain only one representative variable for each group. The selected variable should significantly explain variation in the diatom assemblage and be statistically independent in order to be a potential variable for transfer function development (Fallu, 1998). To determine the appropriate ordination method, gradient length was assessed using detrended correspondence analysis (DCA) on Hellinger‐transformed taxa to reduce the influence of rare taxa (Legendre & Gallagher, 2001). The results suggested that redundancy analysis (RDA) should be used (gradient length < 2SD). For each group of correlated variables, a series of RDAs and partial RDAs were performed using forward selection and Monte Carlo permutation tests (999 unrestricted permutations, *p* ≤ 0.01) to identify variables to represent the correlated group for the final RDA (Fallu, 1998; Weckström & Juggins, 2005). If more than one variable was chosen by the forward selection, each variable was tested against the others as co‐variables to verify whether the explained variance was independent. The variable explaining a higher part of the variance was retained if variables were dependent; otherwise, all independent variables were retained for the final RDA (Fallu, 1998). Partial RDAs were then used with all final retained variables to determine the percentage of variation explained in the assemblages by each parameter, and a permutation test (999 unrestricted permutations, *p* ≤ 0.01) was performed to verify their significance (Birks, 2010). The arrowhead indicated where the maximum variance for a variable was, and the arrow length was proportional to the variance explained by the variable (ter Braak & Verdonschot, 1995). The position of the sites on the figure showed the relationship between them. If two sites were next to each other, they had similar water conditions. The RDA analyses aimed to identify environmental variables suitable for the development of inference models.

All significance analyses were tested using the anova function from the vegan package in R. Finally, the eigenvalues (λ) and the ratio of λ_1_/λ_2_ were examined. A high ratio (>0.5), in which the variation explained by the first constrained axis was higher compared to the second non‐constrained axis, indicated that the variable significantly explained the variation in the assemblages and could be used for calibration (Fallu & Pienitz, 1999; Juggins, 2013; Vélez‐Agudelo et al., 2021).

### Calibration model development

Several methods for transfer functions were tested to identify the best one for our data. Different types of weighted‐averaging (WA) analyses were performed: classical (WA_cla_) and inverse weighted‐averaging (WA_inv_) deshrinking regressions, tolerance down‐weighting (WA_tol_), and weighted‐averaging partial least squares (WAPLS). Everything was tested using Hellinger‐transformed diatom data and non‐transformed data to explore model performance and to keep a coherence with the RDA analyses. The model with the highest *r* ^2^, the lowest root mean square error of prediction (RMSEP), and the lowest maximum bias was selected to obtain the best inference model (Juggins & Birks, 2012). The jack‐knife method (leave‐one‐out) was used to calculate the predictive ability of each model. All models were performed with the Rioja package in R (Juggins, 2023). For the chosen calibration models, scatter plots showing the correspondence between the observed and diatom‐inferred parameters were generated, and indicator species were identified using the multipatt function from the Indicspecies package (De Cáceres & Legendre, 2009). Optima and tolerances were extracted using the op_calculate function from the optimos.prime package (Sathicq & Nicolosi Gelis, 2020). Finally, transfer functions were tested for autocorrelation using the rne function from the paleoSig package (Telford, 2013). This function compares model performance during cross‐validation under three conditions: when sites are deleted randomly from the training set, when geographically close sites are excluded, and when environmentally similar sites are deleted. There is no autocorrelation if the effect on performance by random exclusion is similar to geographical exclusion. Similar species assemblages and environmental conditions at sites close to each other can create a lack of independence between the sites and lead to wrong estimations of transfer function performance (Telford & Birks, 2009).

## RESULTS

### Environmental framework

**FIGURE 2:**
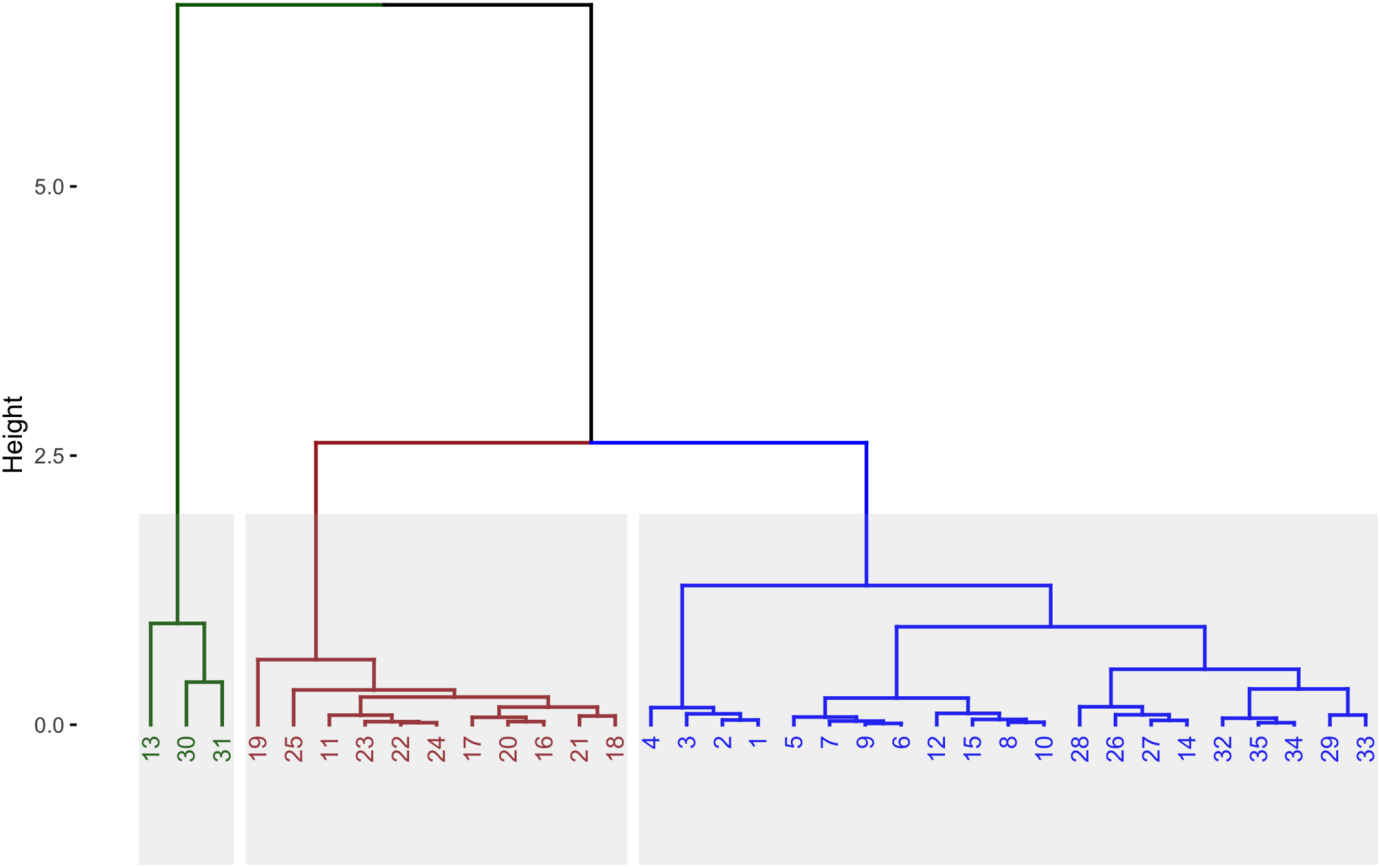
Hierarchical clustering showing three different clusters of sites, according to the water parameters. Cluster 1 in green (River cluster), cluster 2 in brown (BSI cluster), and cluster 3 in blue (W‐E cluster).

Cluster analyses based on the environment revealed that the transect was divided into three groups (Figure 2). The first represented the river cluster (sites 13, 30, and 31), grouping the sites near the main rivers, Ste‐Marguerite (site 13) and Moisie (sites 30 and 31). The second grouped sites 16–25 that were in the BSI and site 11 in the western part of the transect (BSI cluster). The third included sites located outside of the Bay (W‐E cluster).

Water transparency was lower in the BSI cluster, characterized by a minimum turbidity of 1.55 FTU and a maximum of 2.83 FTU. Additionally, the Secchi depth readings revealed lower values in the BSI cluster (mean of 6.12 m) and River cluster (a mean of 8.4 m) compared with sites in the W‐E cluster (13.59 m). Surface temperatures varied from 9.9°C (site 31) to 13.01°C (site 20), with temperatures in the BSI cluster showing a higher mean of 12.29°C, ranging between 11.59 and 13.01°C, whereas temperatures averaged 11.27°C in the W‐E cluster and 10.95°C in the River cluster. Salinity ranged from 22.5 (site 13) to 29.3 (site 35), with the lowest mean observed in the River cluster (24.6), whereas salinity remained relatively consistent across the other sites. Along the transect, pH exhibited minimal variation between 8.1 and 8.9. Total phosphorus concentrations spanned from 25 at site 9–235 μg · L^−1^ at site 28 in the W‐E cluster. Concerning dissolved silica concentrations, values ranged from 0 at site 34 in the W‐E cluster to 1.5 mg · L^−1^ at site 30 in the River cluster, with concentrations being higher at river sites, with a mean of 1.1 mg · L^−1^ compared to 0.1 and 0.2 mg · L^−1^ in the BSI and W‐E clusters, respectively. Ions (K, Ca, Na, Mg, Cl, and SO_4_) all showed a similar trend, wherein concentrations dropped at River cluster sites (13, 30, and 31) and were lower in the BSI cluster. As an example, Mg showed means of 500.77 in the River cluster, 989.91 in the BSI cluster, and 995.36 mg · L^−1^ in the W‐E cluster.

The average concentrations of Fe and Mn were 50.1 and 4.9 μg · L^−1^, respectively. Iron ranged from <20 to 519 μg · L^−1^; however, 70% of the sites were below the detection limit of 20 μg · L^−1^. Three sites had high concentrations of Fe, and site 19, located next to industries and docks on Pointe‐Noire, had a particularly high concentration of Fe (519 μg · L^−1^) followed by neighboring site 18 (137 μg · L^−1^). Site 5, in Port‐Cartier, had an Fe concentration of 126 μg · L^−1^. Manganese concentrations ranged between 2 and 16 μg · L^−1^, with the highest concentrations recorded at sites 17 (16 μg · L^−1^), 18 (9.5 μg · L^−1^), and 19 (10 μg · L^−1^), all located around the Pointe‐Noire Terminal. On average, concentrations were higher in the BSI cluster with a mean of 7.9 μg · L^−1^, as opposed to a mean of 3.5 μg · L^−1^ in the River and W‐E clusters.

### Diatom assemblages

A total of 735 diatom taxa were identified in the 35 samples, but only 74 taxa (Figure S1–S11) met our cut‐off criterion (relative abundance ≥1% in at least two sites). However, these 74 taxa represented 82% of the total diatoms counted. The high initial number of taxa was partially attributed to the detailed separation of several girdle views based on morphology and ecology, as well as the subdivision of *Chaetoceros* spp. Most of the taxa belonged to the genera *Cocconeis*, *Navicula*, and *Achnanthes*. Species richness in the surface sediment assemblages varied from 22 to 54 per sample and was on average 42 taxa. A total of 55 benthic taxa were identified, compared to only 19 planktonic taxa.

**FIGURE 3:**
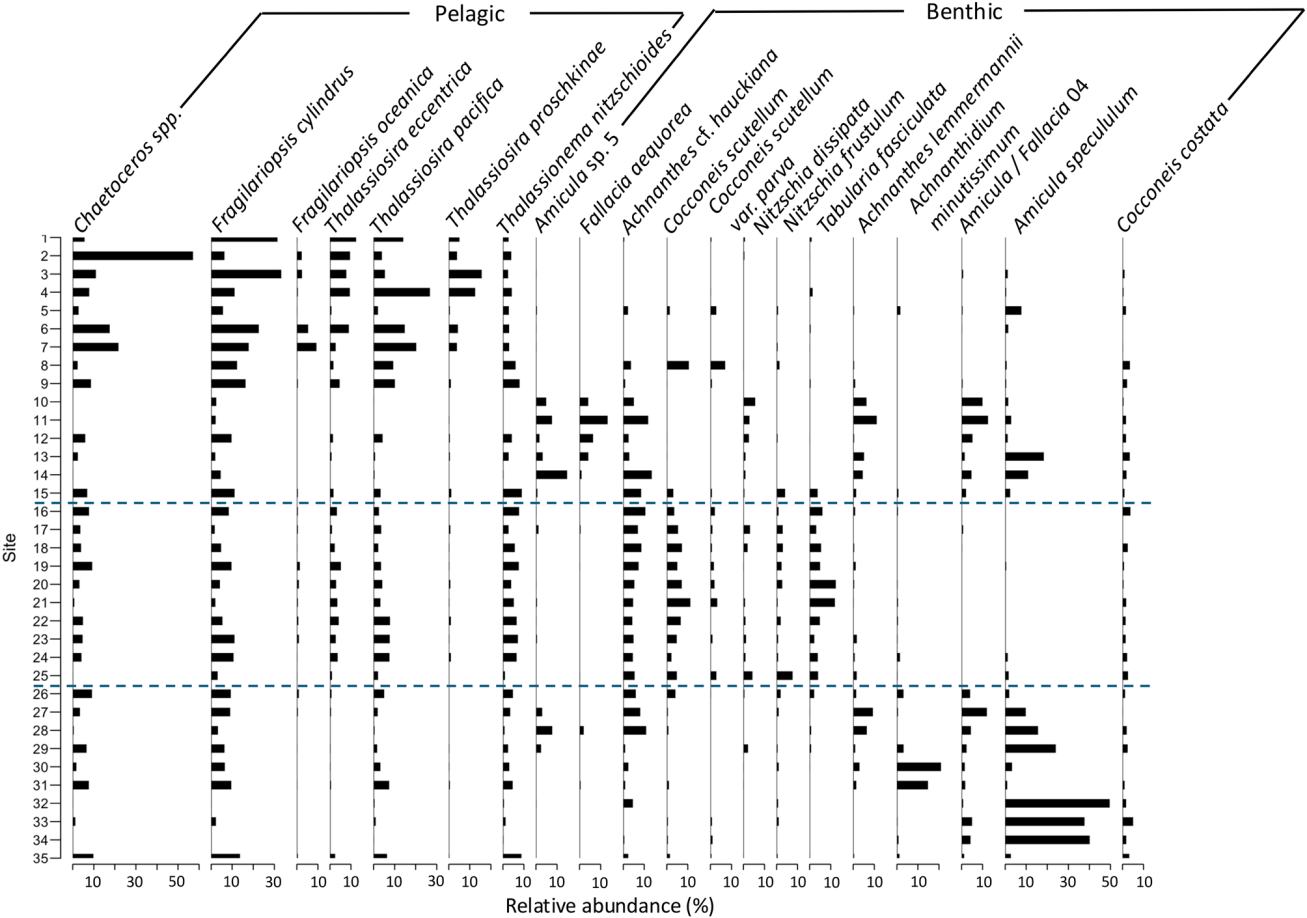
Most common taxa across the transect sites with at least 5% relative abundance, ordered according to the site at which they reached peak abundance, to better observe evolution assemblage throughout the transect from west to east. Dashed lines delimit the sites located inside the BSI (16–25).

*Fragilariopsis cylindrus* was the most common species throughout the transect, followed by *Thalassiosira pacifica* and *Thalassionema nitzschioides*. The west group was dominated by *F. cylindrus*, representing 15% of the assemblages, *Thalassiosira pacifica* (10%), and *Chaetoceros* sp.3 (8%). Meanwhile, *Amicula specululum* (31%) was the most abundant species in the eastern part of the transect. Some species, such as *Amicula* sp. and *Achnanthes lemmermannii*, were absent within the BSI but occurred in abundance at sites on both sides of the bay. Diatom composition in the River cluster showed a higher percentage of freshwater taxa, such as *Achnanthidium minutissimum*, which represented an abundance of 14% at these sites. Some taxa seemed to be more abundant in certain areas of the transect as shown in Figure 3. *Cocconeis scutellum*, *Nitschia frustulum*, and *Tabularia fasciculata* were mostly located in the BSI, which was dominated by *F. cylindrus* (8%), *Achnanthes* cf. *hauckiana* (8%), and *Thalassionema nitzschioides* (6%). The BSI assemblage was mostly composed of benthic and particularly epiphytic taxa, such as *Cocconeis scutellum* and *Tabularia fasciculata*. Notably, *Grammatophora marina* and *G. oceanica* were unique to sites within the BSI. Pelagic taxa, in contrast, were more abundant in the western part of the transect. Overall, the transect was dominated by coastal marine to brackish diatoms; some freshwater taxa were also observed at every site but were more abundant at river mouths.

**FIGURE 4:**
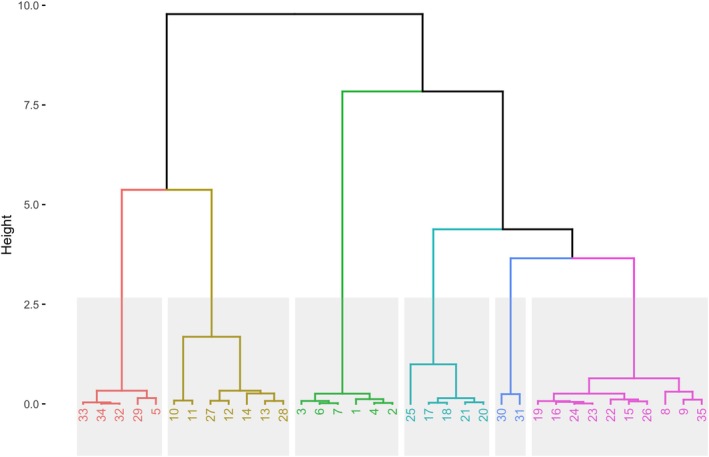
Hierarchical clustering showing six different clusters of the sampling sites, based on diatom assemblages. Cluster 1 in red, cluster 2 in gold, cluster 3 in green, cluster 4 in turquoise, cluster 5 in blue, and cluster 6 in pink.

The cluster analysis performed with diatom assemblages (Figure 4) did not present the same groups as the clusters obtained using only the environmental parameters (Figure 2). Sites 30 and 31, located near the mouth of the Moisie River, were grouped into one cluster (blue); however, site 13, located at the mouth of the Ste‐Marguerite River, was in a different cluster (gold). Sites 16–25, previously part of the BSI cluster, were separated into two different groups (turquoise and pink clusters).

### Variable selection and redundancy analyses

**FIGURE 5:**
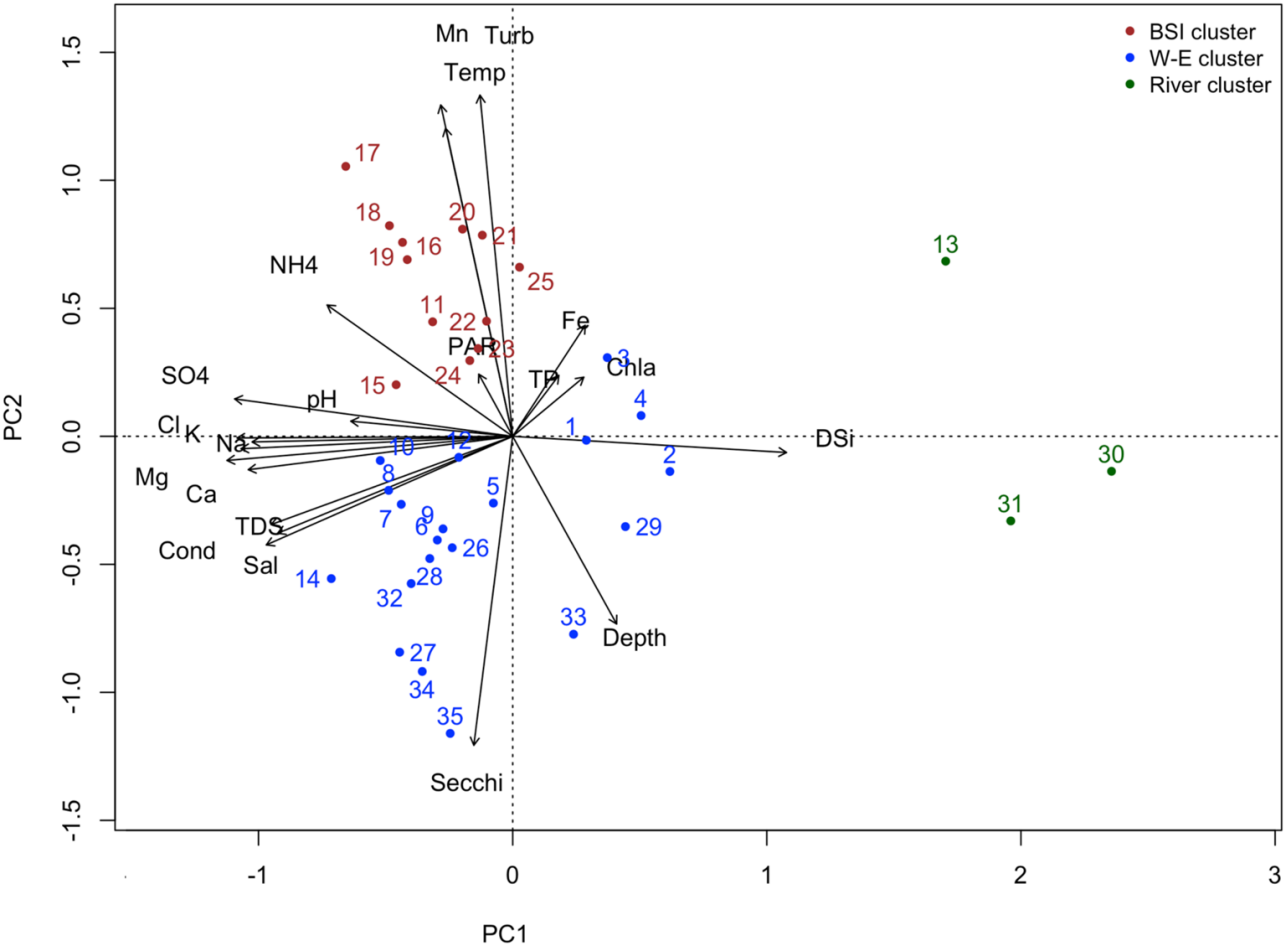
Principal component analysis (PCA) ordination diagram showing the relationships between the measured environmental variables (black arrows) and sites. Sites appear on the figure with colors according to their cluster.

The correlation structure between environmental data and sampling sites was summarized as a PCA biplot (Figure 5) and Pearson correlation matrix (Figure S12). Sites from the BSI cluster appeared to have water conditions that were more turbid, with higher temperatures and higher concentrations of Mn, as opposed to the W‐E cluster. Sites in the River cluster were all located at the right of the figure (Figure 5), indicating that they had different water conditions from the rest of the sites. These sites stood in opposition to the salinity arrow. Arrows pointing in the same direction and close to each other show a collinearity. According to the correlation matrix and the PCA, three groups of highly correlated variables were identified: (1) variables reflecting salinity—salinity, TDS, and conductivity; (2) variables reflecting water chemistry—Cl, Na, K, SO_4_, DSi, and Mg; and (3) variables reflecting mainly physical parameters—Secchi, temperature, turbidity, and Mn. Regarding species selection tested within the RDAs, an initial RDA conducted with both planktonic and benthic taxa did not yield any variable suitable for transfer function development, as none satisfied the λ_1_/λ_2_ > 0.5 criterion. For this reason, only benthic taxa, which were overall more abundant and diverse than planktonic taxa, were selected from the initial 74 taxa, resulting in a total of 55 benthic taxa used for the subsequent analyses.

**FIGURE 6:**
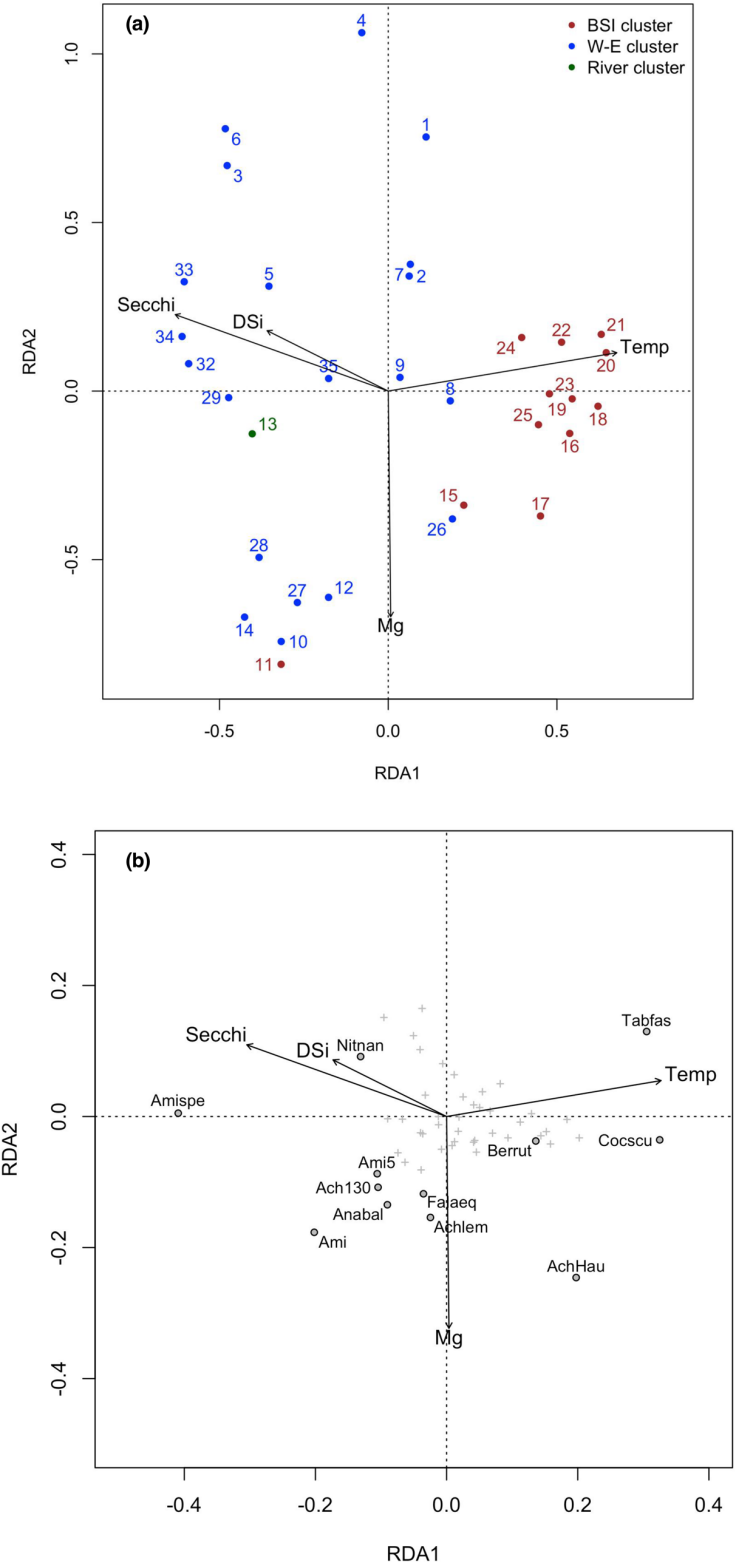
Redundancy analysis (RDA) showing the relationships between sites (a) and species (b) between the four selected environmental variables. The species selected have a relative abundance >5% in at least two sites (Nitnan = *Nitzschia nanodissipata*; Amispe = *Amicula specululum*; Ami5 = *Amicula* sp.5; Ach130 = *Achnanthes* sp.130; Anabal = *Anaulus balticus*; Ami = *Amicula*/*Fallacia* sp.4; Falaeq = *Fallacia aequorea*; Achmen = *Achnanthes lemmermannii*; Achhau = *Achnanthes* cf. *hauckiana*; Cocscu = *Cocconeis scutellum*; Berrut = *Berkeleya rutilans*; Tabfas = *Tabellaria fasciculata*).

For the first correlated group, none of the variables explained a significant part of the variance, but salinity was kept as a condition in the final RDA because of its ecological importance for diatoms. Forward selection and independence tests found DSi and Mg could explain a significant part of the variance in the second group. Turbidity was removed from the third group, as it is the same kind of measure as the Secchi disk, but the latter has better reproducibility. Secchi measures and temperature were selected after analysis. Depth, TP, PAR, Chl *a*, salinity, Fe, and NH_4_^+^ did not explain a significant part of the variance but were kept as covariates in the final RDA due to their ecological importance. The final RDA (Figure 6a,b) was performed using the four selected variables (Table 2) and the seven covariates and included 55 benthic taxa. All sites were included except sites 30 and 31, which were excluded as outliers due to their distinct environmental and assemblage characteristics, as confirmed by PCA (Figure 5) and cluster analyses (Figures 2 and 4). All the variables, including covariates, explained 48% of the total variance in the benthic assemblage. The four constrained variables explained 28.6%, and the covariates explained 23.6% of the variance. Temperature explained the most variance (14.62%), followed by Secchi (12.97%), DSi (6.91%), and Mg (6.23%). Both temperature and Secchi met the λ_1_/λ_2_ > 0.5 criterion; however, only temperature was selected to develop a transfer function. Temperature has been more commonly used in other studies, facilitating comparison. Additionally, these two variables were strongly correlated and thus likely to reflect similar environmental gradients.

**TABLE 2:** Variance explained for each variable used in the RDA, their significance, and the eigenvalues ratio test for developing transfer function.

Variable	Variance explained (%)	RDA1 (%)	PC1	*λ*_1_/λ_2_	*p* value
Temperature	14.62	7.83	8.81	0.89	0.001
Secchi	12.97	6.95	10.01	0.69	0.001
DSi	6.91	3.70	13.57	0.27	0.05
Mg	6.23	3.34	14.93	0.22	0.05

### Diatom‐based inference model for temperature

#### Calibration model for temperature

**FIGURE 7:**
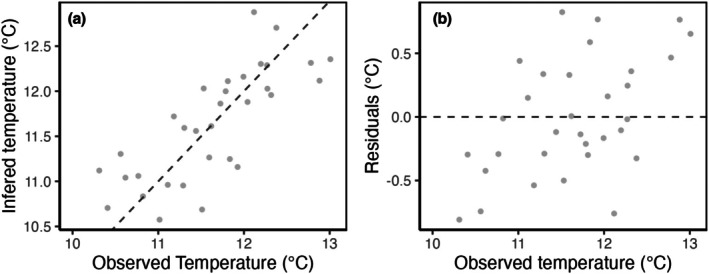
Scatter plot showing the regression (a) and residuals (b) analysis of diatom‐inferred temperature based on a WA‐PLS (3) with Hellinger‐transformed data.

To identify the best calibration model for reliable inferences in the study area, several WA and WAPLS models were developed using untransformed and square‐root‐transformed taxa data (Table 3). The different analyses revealed that the three‐component WAPLS with Hellinger‐transformed taxa had the best performance, with the highest *r* ^2^_jack_ (0.59), the lowest RMSEP (0.45°C), and a maximum bias of 0.63°C. There was, however, relatively low variation along the gradient overall (10.3–13°C), and the lowest value of 9.9°C was recorded at site 31, which was excluded from the model. No trend was observed in the residuals. The performance statistics and scatter plots collectively suggested that the model could provide reliable temperature predictions (Figure 7). The training set included 55 taxa and 32 sites. Sites 30 and 31 were excluded based on an RDA, and site 7 was removed from the transfer function due to a larger difference between its observed and predicted values than for the other sites. This improved the model performance, increasing the cross‐validated *r* ^2^ from 0.52 to 0.59.

**TABLE 3:** Performance of apparent and cross‐validated (leave‐one‐out) results of several temperature calibration models with and without (in italics) Hellinger data transformation.

	Apparent RMSE		Apparent *r* ^2^		RMSEP		*r* ^2^ jack		Average bias		Max bias	
WA_inv_	0.45	*0.45*	0.59	*0.59*	0.51	*0.52*	0.46	*0.44*	0.01	*0.00*	0.92	*0.96*
WA_cla_	0.58	*0.58*	0.59	*0.59*	0.64	*0.65*	0.50	*0.47*	0.01	*0.00*	0.67	*0.74*
WA_tol‐inv_	0.41	*0.41*	0.65	*0.66*	0.50	*0.53*	0.49	*0.44*	0.00	*−0.01*	0.92	*0.97*
WA_tol‐cla_	0.51	*0.50*	0.65	*0.66*	0.58	*0.60*	0.52	*0.47*	−0.00	*−0.01*	0.74	*0.82*
WAPLS (1)	0.45	*0.45*	0.59	*0.59*	0.51	*0.53*	0.46	*0.45*	0.01	*0.02*	0.92	*0.93*
WAPLS (2)	0.23	*0.27*	0.89	*0.85*	0.49	*0.45*	0.54	*0.59*	0.06	*0.03*	0.69	*0.71*
WAPLS (3)	0.21	*0.23*	0.91	*0.89*	0.49	*0.45*	0.54	*0.59*	0.03	*0.00*	0.65	*0.63*

**FIGURE 8:**
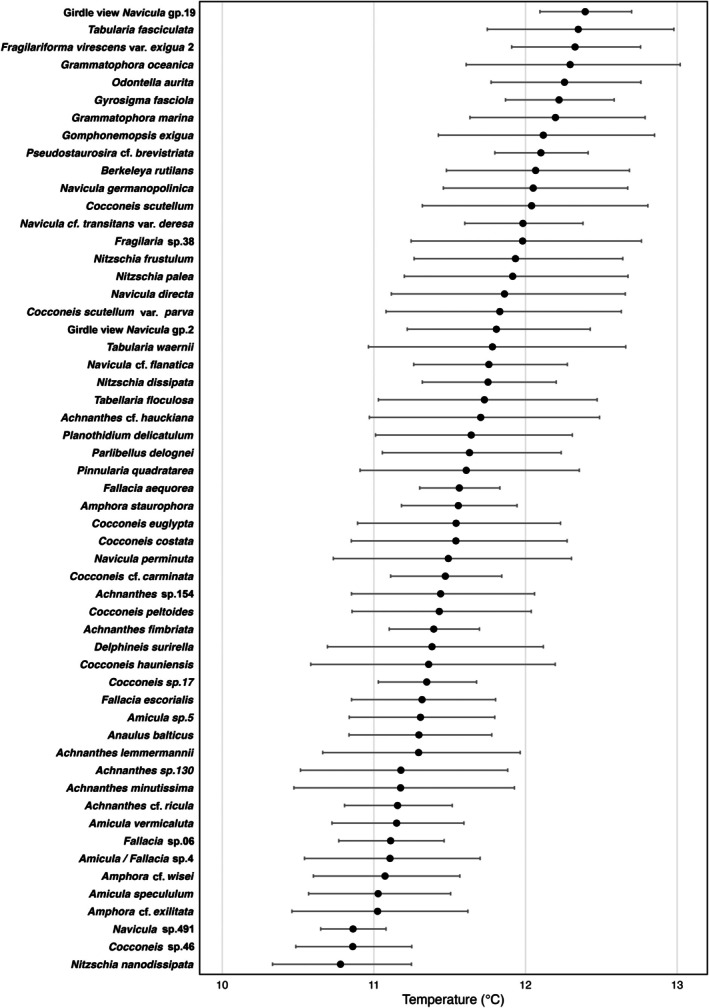
Estimates of taxa optima and tolerances to temperature.

The taxa with the highest temperature optima (Figure 8, Table S1)—Girdle view *Navicula* gp.19 (12.39°C), *Tabularia fasciculata* (12.35°C), *Fragilariforma* var. *exigua 2* (12.33°C), and *Grammatophora oceanica* (12.29°C)—were mostly located in the BSI, where temperatures were higher than in other areas of the transect. The indicator species analyses determined these taxa to be good indicators of temperature between 12 and 13°C as were the taxa *Gomphonemopsis exigua*, *Gyrosigma fasciola*, and *Berkeleya rutilans*, also particularly abundant in the BSI cluster. *Nitzschia nanodissipata*, mostly observed in the W‐E cluster, had the lowest optimum of 10.78°C and could be an indicator of temperatures between 10 and 11°C.

#### Autocorrelation test

**FIGURE 9:**
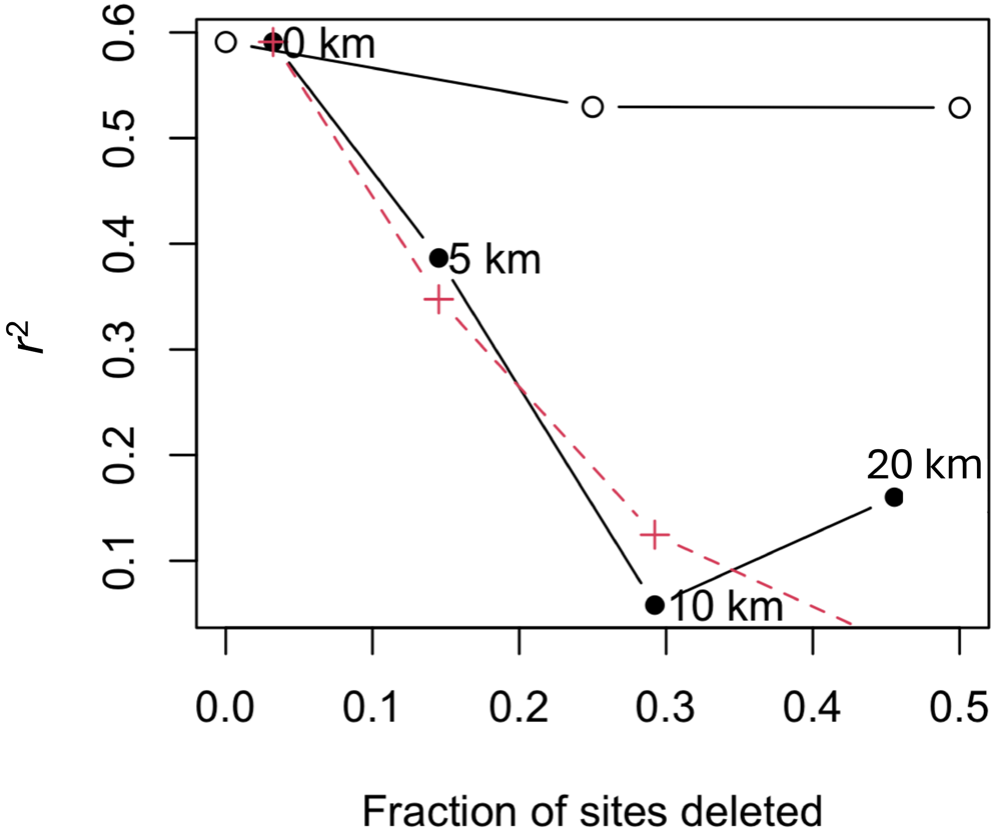
Effect on transfer function *r* ^2^ of deleting sites at random (open circles), from the geographical neighborhood of the test site (filled circles), and from environmentally most similar sites (red crosses) during cross‐validation. The WAPLS (3) is used.

The effects of spatial structure in the training set using a random, neighbor, environment deletion analysis (RNE) showed that random removal of sites had a modest effect on the transfer function performance. The full model *r* ^2^ decreased from 0.59 to 0.53 when 50% of the samples were removed randomly (Figure 9). However, excluding geographically close sites resulted in a decline of the model performance: *r* ^2^ dropped below 0.1 after sites within 10 km were removed. A similar decline in performance was observed when environmentally similar sites were removed. This result indicated that there is spatial autocorrelation among sites. At a 20‐km exclusion for close sites, the model recovered some predictive power but was still low, with an *r* ^2^ = 0.16. This suggested that the transfer function could capture some ecological information even with spatial autocorrelation. Given how some assemblages were similar at sites in the same zones, such as in the BSI cluster, autocorrelation was expected.

## DISCUSSION

Temperature and Secchi depth were the most significant variables explaining most of the variation in the benthic diatom assemblages in our study area that could be used for transfer functions. However, only temperature was used to develop a calibration model for future environmental reconstructions. These two variables being highly correlated, the results of either would have generated similar changes.

### Calibration model and limits

Temperature is an important variable known to have an influence on diatoms (Leventer et al., 2012), an influence that has been demonstrated with respect to freshwater environments (e.g., Pienitz et al., 1995; Weckström et al., 1997). In our study, temperature explained 14.6% of the variance of the assemblages, and the temperature was higher inside the Bay. This could be explained by the fact that the Bay is semi‐enclosed by an archipelago (Carrière & Le Hénaff, 2018). The variability of diatom assemblages from the intertidal zone of the BSI was also significantly influenced by temperature (Arseneault et al., 2023). The three‐component WAPLS (3) calibration provided the best performance for the temperature model out of the seven models tested. The model, however, had a moderate predictive power, with an *r* ^2^ = 0.59, which implied that only 59% of the observed temperature would be explained by the model and that there was still some unexplained variability, probably due to other environmental factors. Compared to other studies in the same kind of environment, this model has a weaker predictive power and a smaller gradient (Table 4). This limited gradient may pose challenges to model development. For example, Lapointe (2000a) was unable to develop a transfer function in the Gulf of St. Lawrence, partly due to an insufficient number of sites (*n* = 41) to capture the variability of the region.

**TABLE 4:** Examples of diatom‐based temperature transfer function performances from other studies around the world.

Region	Nb. samples	Gradient (°C)	Model type	*r*_2 jack/boot_	RMSEP (°C)	References
Sept‐Îles	33	10.3–13	WAPLS (3)	0.59	0.45	This study
Disko Bay, West Greenland	35	1–6	WAPLS (5)	0.881	0.395	Krawczyk et al. (2017)
New Zealand	38	12–21	WA‐tol	0.81	1.57	Cochran and Neil (2010)

Several studies have successfully used sea‐surface temperatures for transfer functions in the Arctic, Subarctic (Miettinen, 2018), and Antarctic regions (Zielinski et al., 1998), observing climate changes over time. For instance, a study from Disko Bay in West Greenland applied sea‐surface temperature transfer functions, and the reconstructions identified changes over the last ~11,000 years, despite a relatively short temperature gradient between 1 and 6°C (Krawczyk et al., 2017). Diatom‐based temperature transfer functions, however, often face criticism in limnology. Temperature models are sometimes considered weaker than other variables such as pH or salinity, since temperature is not considered as having as much impact on diatoms as water chemistry. Moreover, many diatom species are cosmopolitan, which could reduce the ecological signal (Anderson, 2000). In marine and coastal environments, transfer functions are further challenged by spatial structures that can cause cross‐validated estimates of the prediction power to be overoptimistic (Telford & Birks, 2009). In our present study, the calibration model was indeed influenced by autocorrelation, a result that was anticipated given the taxa structures observed. The training set was composed of a small sample size of 32 sites, and reducing the number of samples often worsens performance (Telford & Birks, 2009). For the final model, all 32 sites were retained, but any spatial effects must be considered when interpreting paleoreconstructions based on this model, as its predictive power could be uncertain. Diatoms are influenced by numerous interconnected, and often correlated, environmental parameters. In the BSI, a characteristic diatom assemblage was observed, with many species predominantly located in this part of the transect, such as *Cocconeis scutellum*, *Tabularia fasciculata*, *Grammatophora oceanica*, *Berkeleya rutilans*, and *Gomphonemopsis exigua*. Those species were also determined to be good indicators of warmer temperatures. This assemblage, however, could signal a change not only linked to temperature but also linked to other secondary or unmeasured variables. Notably, Juggins (2013) placed temperature in a category of variables with limited direct effect on biology, acting instead as surrogates for multiple other environmental variables. A variable not measured in our study, which could explain a potential direct influence on assemblages and particularly on benthic taxa, would be substrate availability. The BSI currently hosts a large eelgrass meadow (*Zostera marina*) covering 27.42 km^2^ (Paquette, 2018). All the aforementioned species are known to be epiphytic and living on *Zostera* species (Arseneault et al., 2023; Chung & Lee, 2008). The presence of the eelgrass meadow in the BSI could partly explain the distinctive assemblage observed in this area, especially given the affinity of some dominant taxa like *Tabularia fasciculata* and *Cocconeis scutellum* for *Zostera* habitats. However, further investigations into substrate‐type availability along the rest of the transect would be necessary for any conclusions. A solution would have been to develop a model based solely on pelagic taxa, but probably due to their small number (19), no environmental variable met all the criteria required for transfer function development (Figure S13 and Table S2). In light of these results, temperature reconstructions based on the present model must be interpreted with caution, as observed changes may reflect indirect influences rather than direct effects of climate changes.

### Diatom diversity and indicator species

Many of the taxa located along our transect have also been observed in similar environments (Campeau et al., 1999; Lapointe, 2000a; Poulin et al., 1990; Ribeiro, 2010; Snoeijs, 1993). *Fragilariopsis cylindrus*, the most common species, is a marine to brackish‐water planktonic species often located in Arctic and Antarctic cold waters, common in the Baltic Sea, and abundant in the Gulf of St. Lawrence (Snoeijs & Vilbaste, 1994; Lapointe, 2000a; Witkowski et al., 2000). *Thalassiosira pacifica* is another planktonic marine taxon, also abundant in the Gulf and maritime estuary of the St. Lawrence (Bérard‐Therriault et al., 1987; Lapointe, 2000a). *Thalassionema nitzschioides* is a planktonic marine cosmopolitan species (Hasle, 2001) located in the Gulf, the Estuary, and the Baltic Sea (Bérard‐Therriault et al., 1999; Snoeijs & Vilbaste, 1994). *Cocconeis scutellum* and *Tabularia fasciculata* were abundant mainly inside the Bay and in the intertidal zone of the BSI (Arseneault et al., 2023). *Cocconeis scutellum* typically grows on macroalgae, and changes in its abundance along the transect could be linked to a lack of its habitat at other sites (Cooper, 1995), explaining why it was mostly present inside the BSI. *Tabularia fasciculata* is common worldwide in benthic littoral habitats with a large tolerance to salinity and temperature (Kaczmarska et al., 2009; Kociolek, 2011); it is heavily silicified, thus limiting its dispersal over long distances (Kaczmarska et al., 2009), which could also explain its main presence in the Bay. *Amicula specululum* was observed at many sites throughout the transect and was particularly abundant in the eastern part of the transect. The genus *Amicula* was located in brackish to marine sediments, and because of its very small size (<10 μm), it can be difficult to differentiate species under light microscopy, as was the case in this study. *Amicula specululum* has been observed in marine coastal environments, in diverse areas and habitats such as the Baltic Sea and Florida (Gastineau et al., 2022).

Some species are typically observed in cold waters, specifically where there is the presence of sea ice. *Chaetoceros* was quite abundant along the transect in the study area, especially in the western sites, and has often been observed in similar environments. This genus is particularly common in coastal and oceanic waters around the world (Gaonkar et al., 2017; Malviya et al., 2016) and can serve as an indicator of the presence of sea ice. In the bays of Windmill Islands in Antarctica, *Chaetoceros* spp. were abundant and suggested variability in sea‐ice conditions (Cremer et al., 2003). In the Pacific Arctic, *Chaetoceros* spp. and *Thalassiosira* spp. were abundant in areas where sea ice had retreated earlier (Fukai et al., 2021). *Chaetoceros* resting spores, however, are difficult to identify, as it was the case in this study, and valves have often been counted as *Chaetoceros* spp. (Cremer et al., 2003). Since it is not always possible to distinguish the species, it is important to take this into account when using them as potential sea‐ice indicators. Over the recent years, the ice cover in the Sept‐Îles region has been observed to begin later, melt earlier, and decrease in extent and thickness (Allard, 2024; Demers et al., 2018). There was a difference in *Chaetoceros* spp. abundance along the transect: they were more abundant at the western sites than inside the Bay, possibly indicating a difference in sea‐ice variation in these sites. Lapointe (2000b) qualitatively analyzed diatom species from different cores in the Gulf of St. Lawrence and was able to deduce changes in temperatures through time, with *Chaetoceros* spp. being among the indicators. For example, warming surface temperatures, more than 12,000 years ago, were indicated by the appearance of spores and vegetative cells of temperate–water species such as *Chaetoceros* spp. (Lapointe, 2000b). *Fragilariopsis cylindrus*, the most abundant species in the current study, was also observed in greater quantities at the western sites, although it was common throughout the whole transect. This species is usually used in paleoecology studies as an indicator of sea ice (Kang & Fryxell, 1992; Snoeijs & Wecktröm, 2012), but it has also been used as an indicator of cold waters (Oksman et al., 2019). The abundance of *F. cylindrus* in the sediment could reveal warmer conditions and stratified surface waters resulting from ice melting (Cremer et al., 2003). However, the species has also been observed in areas where no ice was present, meaning that it could be mainly an indicator of cold water (von Quillfeldt, 2004). Neither *Chaetoceros* spp. nor *F. cylindrus* were used in the model training set, but they may provide useful information on temperature variability through the evolution of their abundance in sedimentary archives of the region and support inference results.

## CONCLUSIONS

This study has enhanced the understanding of the autecological preferences of coastal diatom taxa in the Sept‐Îles area (Québec, Canada). The region was characterized by distinct assemblages, particularly within the BSI, likely reflecting unique local conditions such as warmer and more turbid waters, as well as the presence of a large eelgrass meadow. The temperature transfer function developed here may offer a valuable tool for exploring recent past environmental variability in this coastal system. Due to spatial autocorrelation and the influence of unmeasured variables such as substrate availability; however, the results should be interpreted with caution. It is challenging to isolate a single driver when assemblages are shaped by the combined effects of multiple environmental variables. The results emphasize the need for careful interpretation and consideration of other environmental factors. For optimal results, the model should be applied specifically within the sampling region and in conjunction with other paleoenvironmental proxies, such as foraminifera or algal and bacterial pigments, to ensure robust and accurate reconstructions. Despite its limitations, this study has reinforced the use of diatoms for detecting ecological or climate‐driven changes in coastal settings. It has also highlighted the importance of continued monitoring and a comprehensive understanding of diatom ecology in response to environmental changes, particularly in the context of accelerating climate change and increasing anthropogenic pressures. This knowledge is crucial for effective environmental management and the preservation of the BSI and similar vulnerable coastal ecosystems.

## AUTHOR CONTRIBUTIONS

**Emilie Arseneault:** Conceptualization (equal); data curation (lead); formal analysis (lead); investigation (lead); methodology (equal); project administration (equal); resources (equal); validation (equal); visualization (lead); writing – original draft (equal); writing – review and editing (equal). **Émilie Saulnier‐Talbot:** Conceptualization (equal); funding acquisition (lead); methodology (equal); project administration (equal); resources (equal); supervision (lead); validation (equal); writing – original draft (equal); writing – review and editing (equal).

## FUNDING INFORMATION

This research was funded via the EcoZone Research Chair, a partnership between Université Laval, the Port of Sept‐Îles, and the Northern Institute for Research in Environment and Occupational Health and Safety (INREST).

## Supporting information


**Figures S1–S11.** Taxa with relative abundance >1%, taxa used for statistical analysis (relative abundance >2% in at least one site) are marked with an asterisk.
**Figure S12.** Pearson correlation matrix of the 21 environmental variables for the 35 sites.
**Figure S13.** Redundancy analysis (RDA) showing the relationships between sites (a) and planktonic species (b) between the three selected environmental variables.
**Table S1.** The 55 benthic species used in the training set for the temperature model, their estimated optima and tolerances.
**Table S2.** Variance explained for each variable used in the RDA, their significance and the eigenvalues ratio test for developing transfer functions.

## Data Availability

The data used for this study will be available in the Borealis database once the manuscript is accepted.
