# Supplementary material for: Species‐environment relationships of coastal diatoms from the Sept‐Îles region, Gulf of St‐Lawrence (Québec, Canada)
Source: J Phycol. 2025 Sep 29;61(6):1681–98. doi: 10.1111/jpy.70094 (PMC12718435; doi:10.1111/jpy.70094)
Supplement: Supplementary file 1 — Figures S1–S11. Taxa with relative abundance >1%, taxa used for statistical analysis (relative abundance >2% in at least one site) are marked with an asterisk. Figure S12. Pearson correlation matrix of the 21 environmental variables for the 35 sites. Figure S13. Redundancy analysis (RDA) showing the relationships between sites (a) and planktonic species (b) between the three selected environmental variables. Table S1. The 55 benthic species used in the training set for the temperature model, their estimated optima and tolerances. Table S2. Variance explained for each variable used in the RDA, their significance and the eigenvalues ratio test for developing transfer functions. [file JPY-61-1681-s001.pdf]

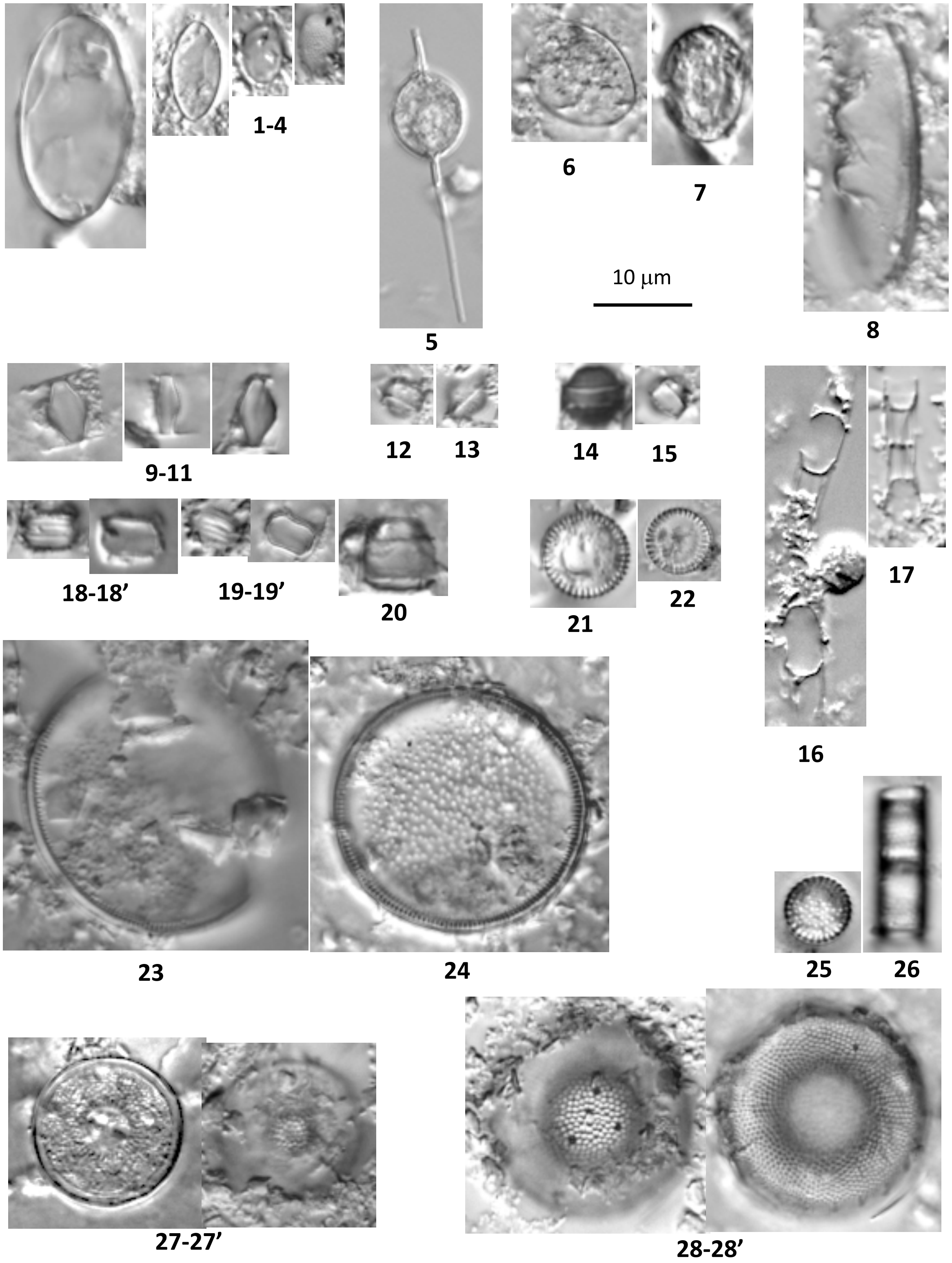

Fig.S1. **1-4:** *Chaetoceros* sp.3*. **5:** *Chaetoceros* cf. *furcellatus**. **6-7:** *Chaetoceros* sp.1. **8:** *Chaetoceros* sp.8*. **9-11:** *Chaetoceros* sp.5*. **12-13:** *Chaetoceros* sp.4*. **14-15:** *Chaetoceros* sp.27. **16-17:** *Skeletonema costatum**. **18-20:** *Chaetoceros* sp.19*. **21-22:** *Cyclotella* cf. *meneghiniana*. **23-24:** *Ehrenbergia* cf. *granulosa*. **25-26:** *Aulacoseira* cf. *granulata*.**27-28’:** *Bacteriosira* cf. *bathyomphala**.

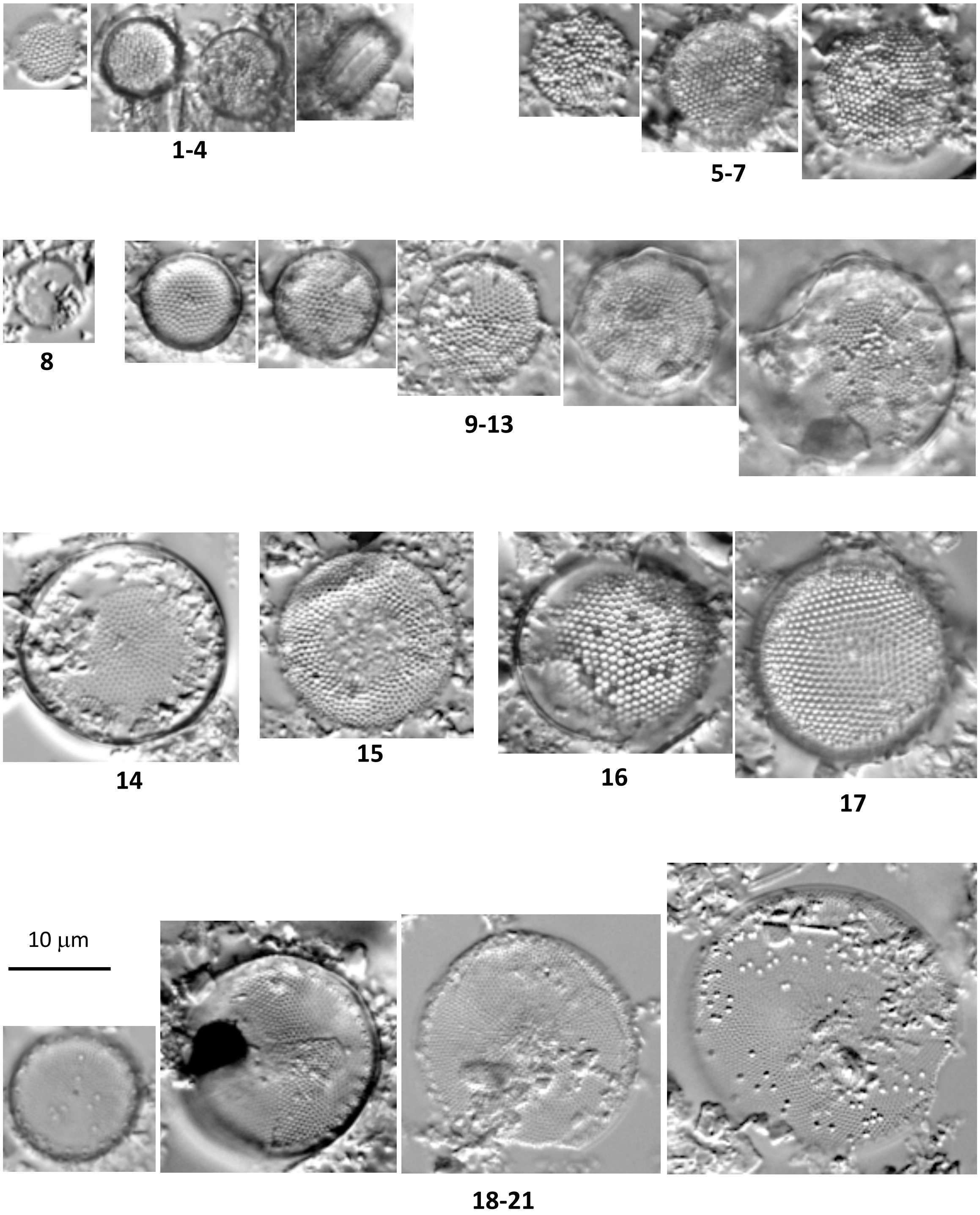

Fig.S2. 1-4: *Thalassiosira proschkinae**. **5-7:** *Thalassiosira* sp.31*. **8:** *Thalassiosira guillardii**. **9-13:** *Thalassiosira pacifica**. **14:** *Thalassiosira* sp.58. **15:** *Thalassiosira hyperborea var pelagica*.* **16-17:** *Thalassiosira eccentrica**. **18-21:** *Thalassiosira hyalina*.*

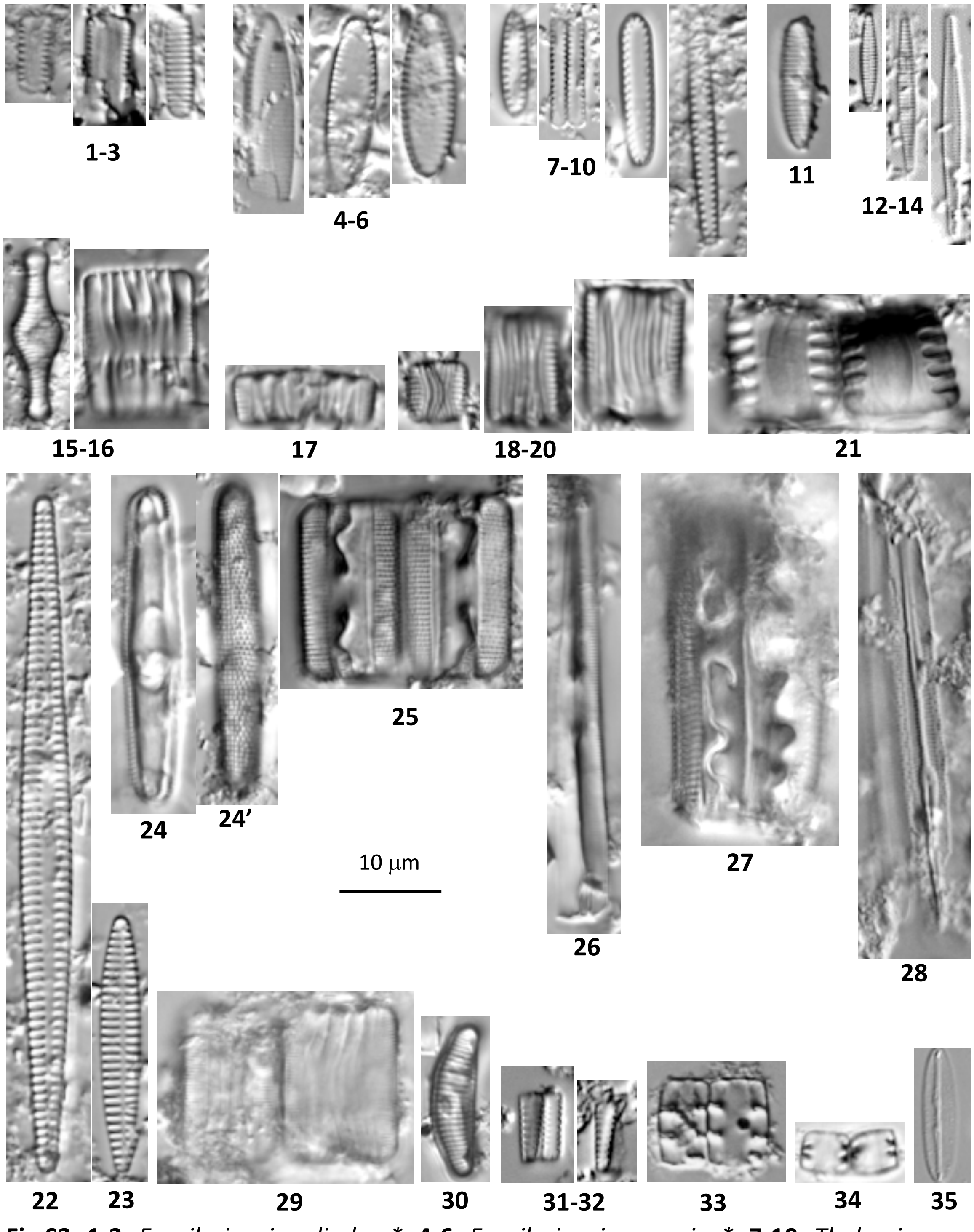

Fig.S3. **1-3:** *Fragilariopsis cylindrus**. **4-6:** *Fragilariopsis oceanica**. **7-10:** *Thalassionema nitzschioides**. **11:** *Fragilaria schulzii*. **12-14:** *Tabularia waernii**. **15-16:** *Tabellaria floculosa**. **17:** *Fragilaria* sp.38*. **18-20:** *Fragilariforma virescens* var. *exigua* type 2*. **21:** *Opephora martyi*. **22-23:** *Tabularia fasciculata**. **24-25:** *Grammatophora marina**. **26:** *Grammatophora oceanica**. **27:** *Grammatophora arcuata*. **28:** *Berkeleya* sp.2. **29:** *Eunotia* sp.71. **30:** *Eunotia incisa*. **31-32:** *Girdle view Fragilaria* sp.59. **33:** *Anaulus balticus**. **34:** *Anaulus minutus*. **35:** *Berkeleya rutilans**.

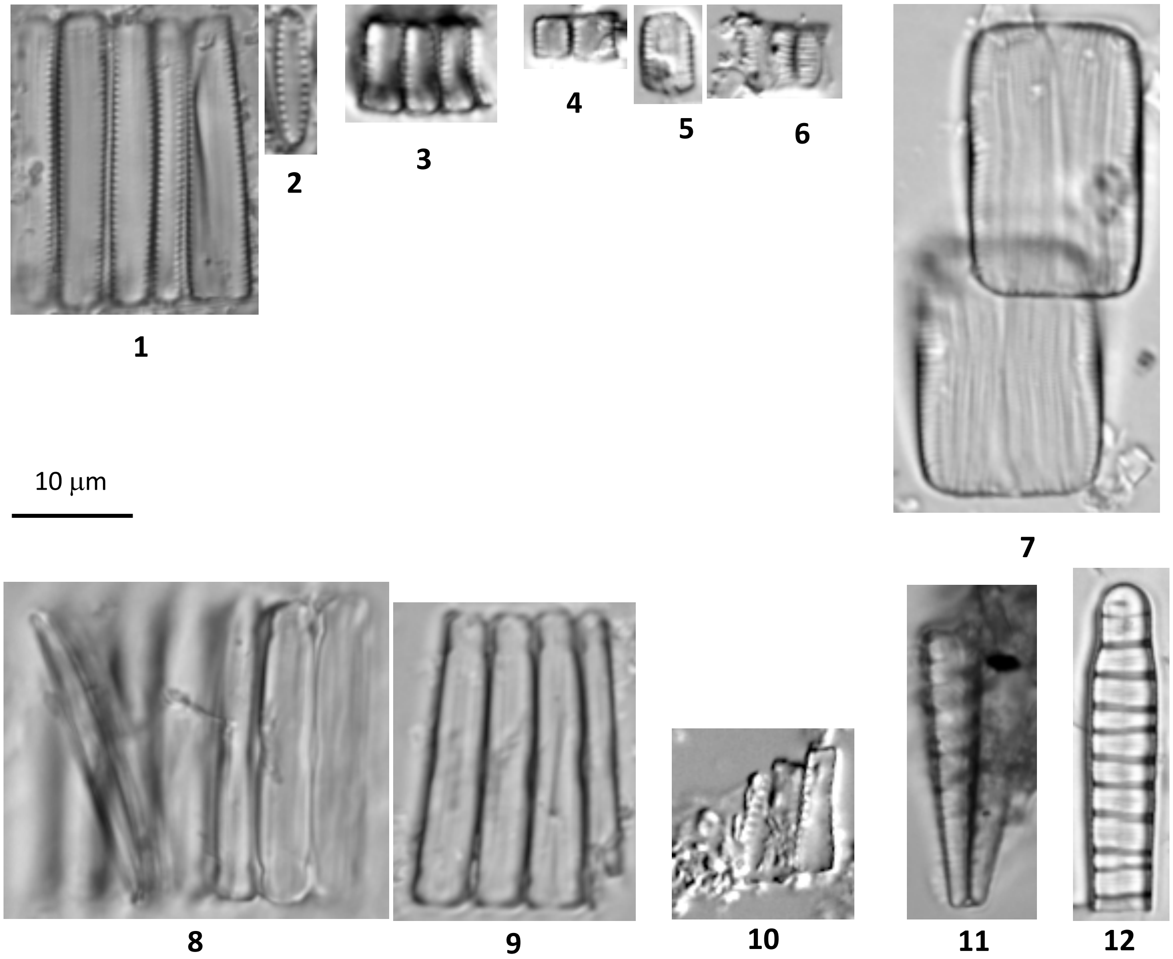

Fig.S4. 1-2: *Pseudostaurosira brevistriata**. **3: Girdle view (442)** *Fragilaria* sp.60. **4-6: Girdle view (314)** *Fragilaria* sp. 61 (Martyana atomus?). **7: Girdle view (461)**. **8-9: Girdle view (464)**. **10: Girdle view (255)** *Fragilaria* sp.62. **11:** *Meridion circulare*. **12:** *Diatoma tenuis*.

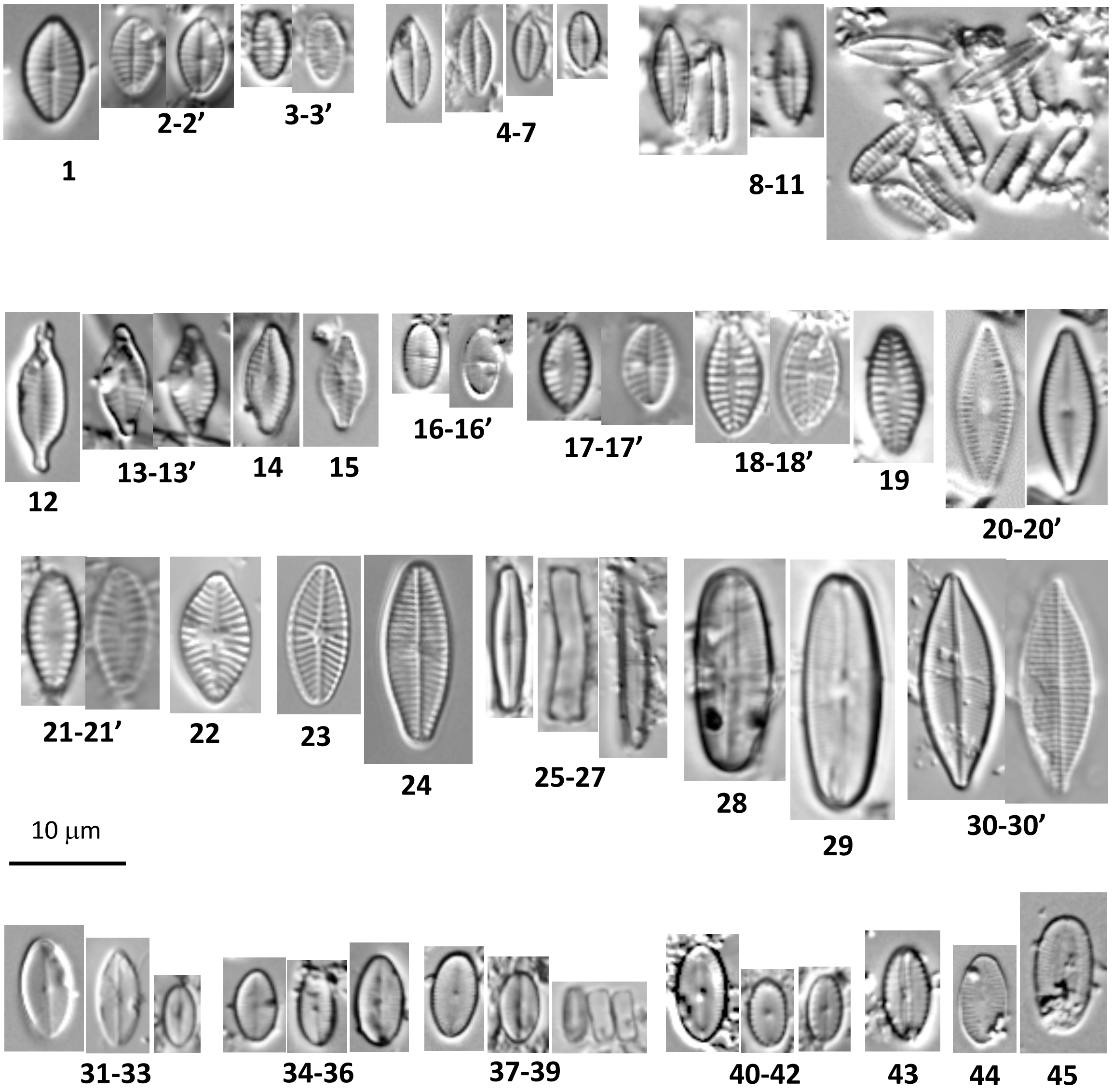

Fig.S5. 1-3’: *Achnanthes* sp.154*. **4-7:** *Achnanthes* cf. *ricula**. **8-11:** *Achnanthes* sp.130*. **12-15:** *Achnanthes lemmermannii**. **16-16’:** *Achnanthes* sp.119. **17-19:** *Achnanthes* cf. *hauckiana**. **20-20’:** *Planothidium diplopunctatum.* **21-22’:** *Planothidium* sp.43. **22:** *Achnanthes delicatula* subsp. *septentrionalis*. **23-24:** *Planothidium delicatulum**. **25-27:** *Achnanthidium minutissimum**. **28-29:** *Achnanthes chlidanos*. **30-30’:** *Achnanthes fimbriata**. **31-33:** *Amicula* sp.5*. **34-36:** *Amicula / Fallacia* sp.4*. **37-39:** *Amicula specululum*.* **40-42:** *Amicula vermiculata*.* **43:** *Fallacia escorialis*.* **44-45:** *Fallacia aequorea*.*

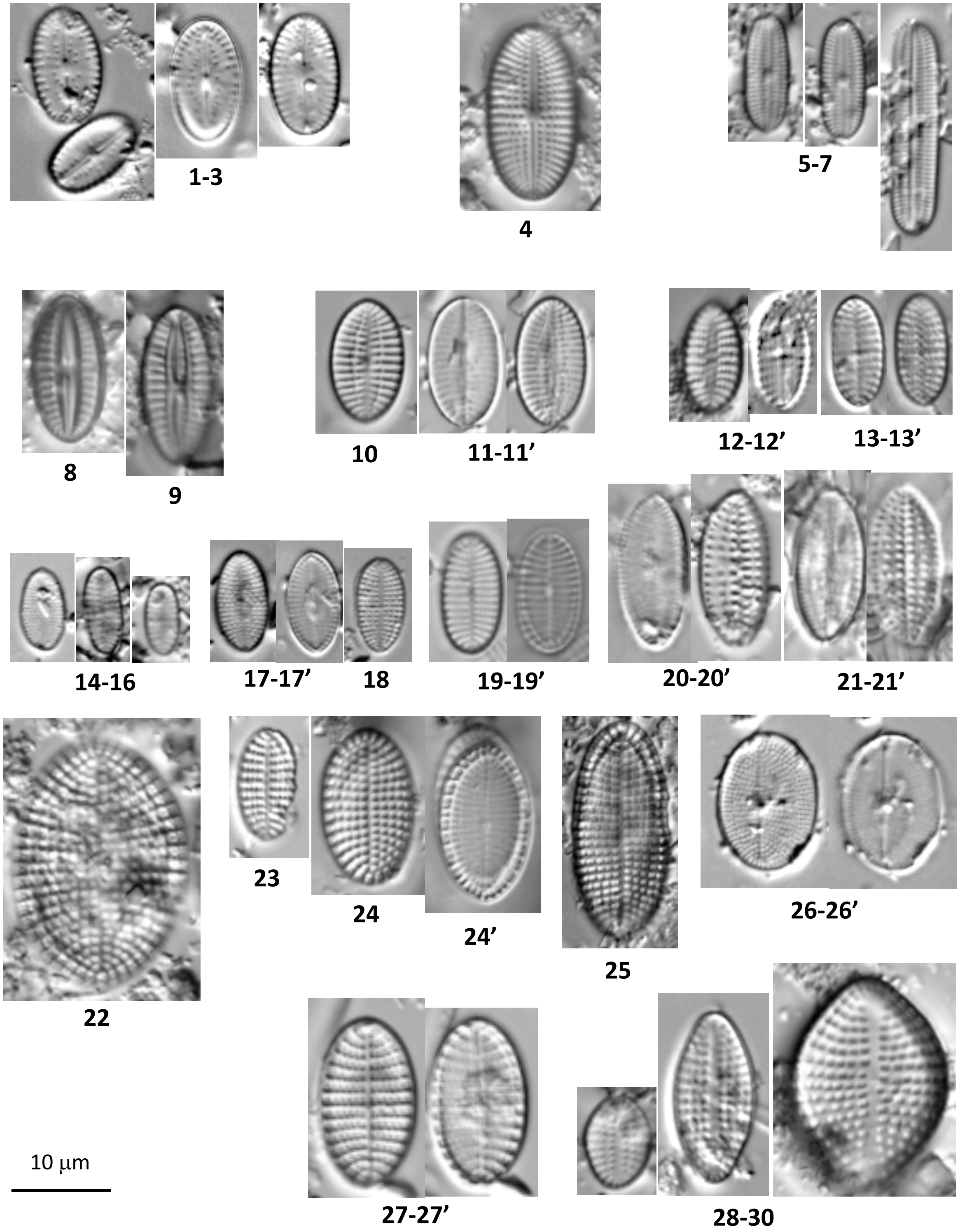

Fig.S6. 1-3: *Fallacia* sp.6*. **4:** *Diploneis* sp.6. **5-7:** *Fallacia sp.30.* **8-9:** *Diploneis reichardtii* var. *tschuktschorum*. **10-11’:** *Cocconeis peltoides**. **12-13’:** *Cocconeis* sp.20. **14-16:** *Cocconeis* cf. *hauniensis**. **17-18:** *Cocconeis euglypta**. **19-19’:** *Cocconeis* sp.46*. **20-21’:** *Cocconeis* sp.17*. **22:** *Cocconeis scutellum**. **23-24’:** *Cocconeis scutellum* var. *parva**. **25:** *Cocconeis scutellum 2.* **26-26’:** *Cocconeis* cf. *carminata**. **27-27’:** *Cocconeis costata**. **28-30:** *Delphineis surirella**.

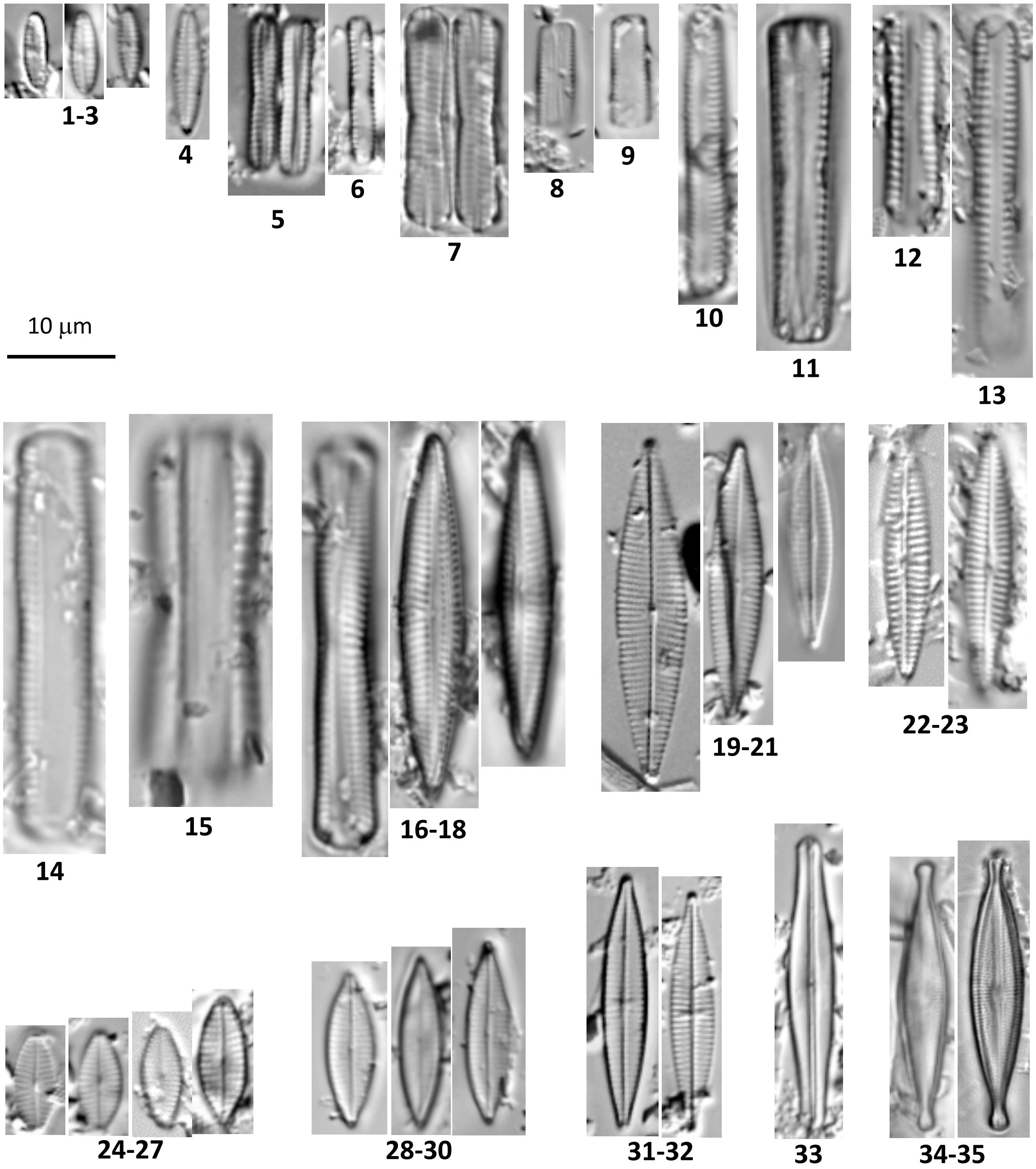

Fig.S7. 1-3: *Navicula perminuta**. **4:** *Navicula* sp.521. **5-6: Girdle view** *Navicula* group 2*. **7: Girdle view** *Navicula* group 21. **8-9: Girdle view** *Navicula* group 18. **10: Girdle view** *Navicula* group 17. **11: Girdle view** *Navicula* sp.445. **12-13: Girdle view** *Navicula* group 19*. **14: Girdle view** *Navicula* sp.437. **15: Girdle view** *Navicula* sp.471. **16-18:** *Navicula* cf. *flanatica**. **19-21:** *Navicula* cf. *transitans* var. *deresa**. **22-23:** *Navicula ramosissima.* **24-27:** *Navicula germanopolinica*.* **28-30:** *Navicula* sp.13. **31-32:** *Navicula*sp.375. **33:** *Kabayasiella* cf. *madumensis.* **34-35:** *Navicula* sp.491*.

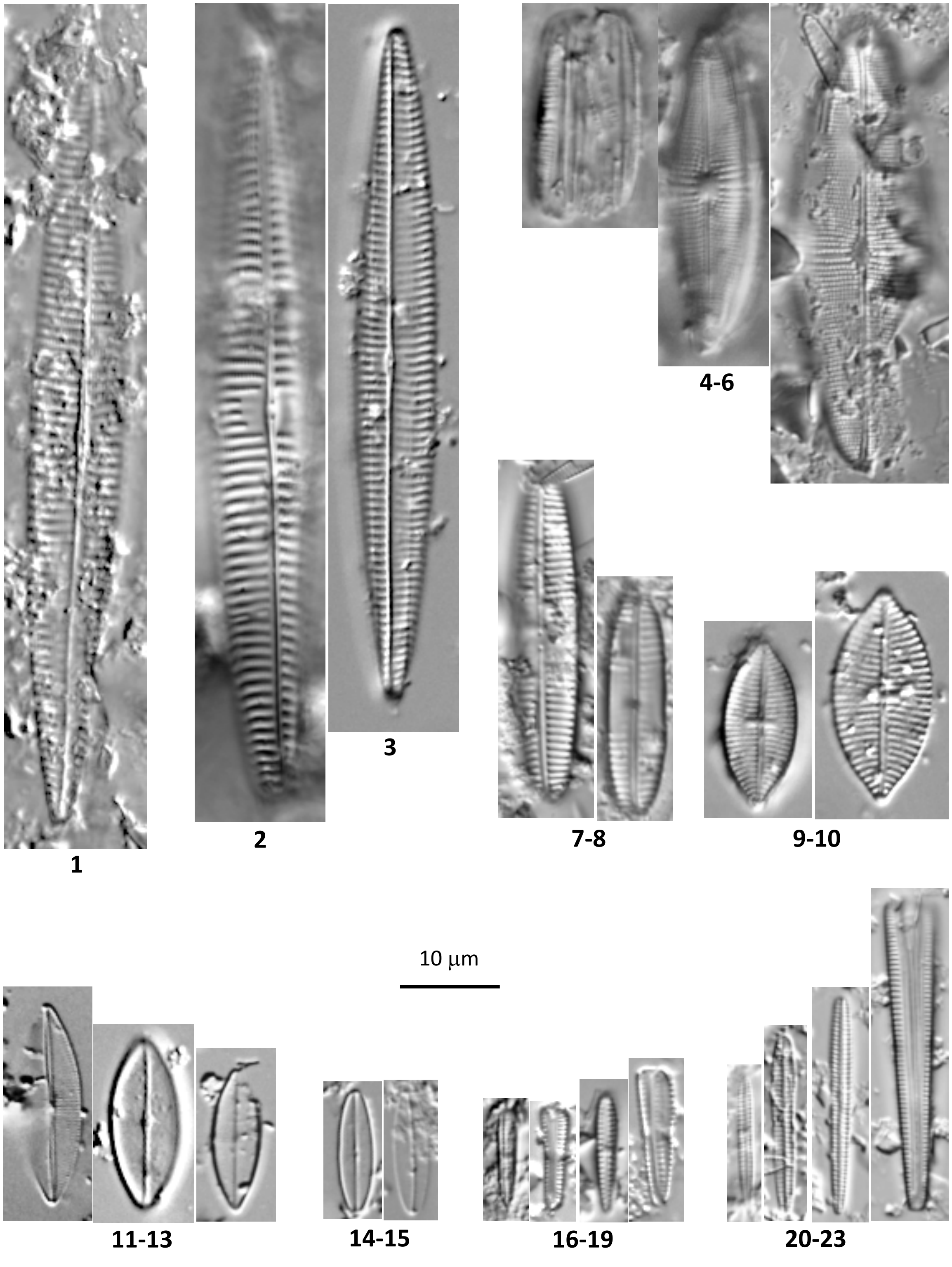

Fig.S8. 1: *Navicula directa* var. *javanica*. **2-3:** *Navicula directa**. **4-6:** *Parlibellus delognei**. 7-**8:** *Pinnularia quadratarea**. **9-10:** *Fogedia* cf. *geisslerae*. **11-13:** *Astartiella bremeyeri*. **14-15:** *Astartiella* sp.2. **16-19:** *Gomphonema pseudexigua*. **20-23:** *Gomphonemopsis exigua**.

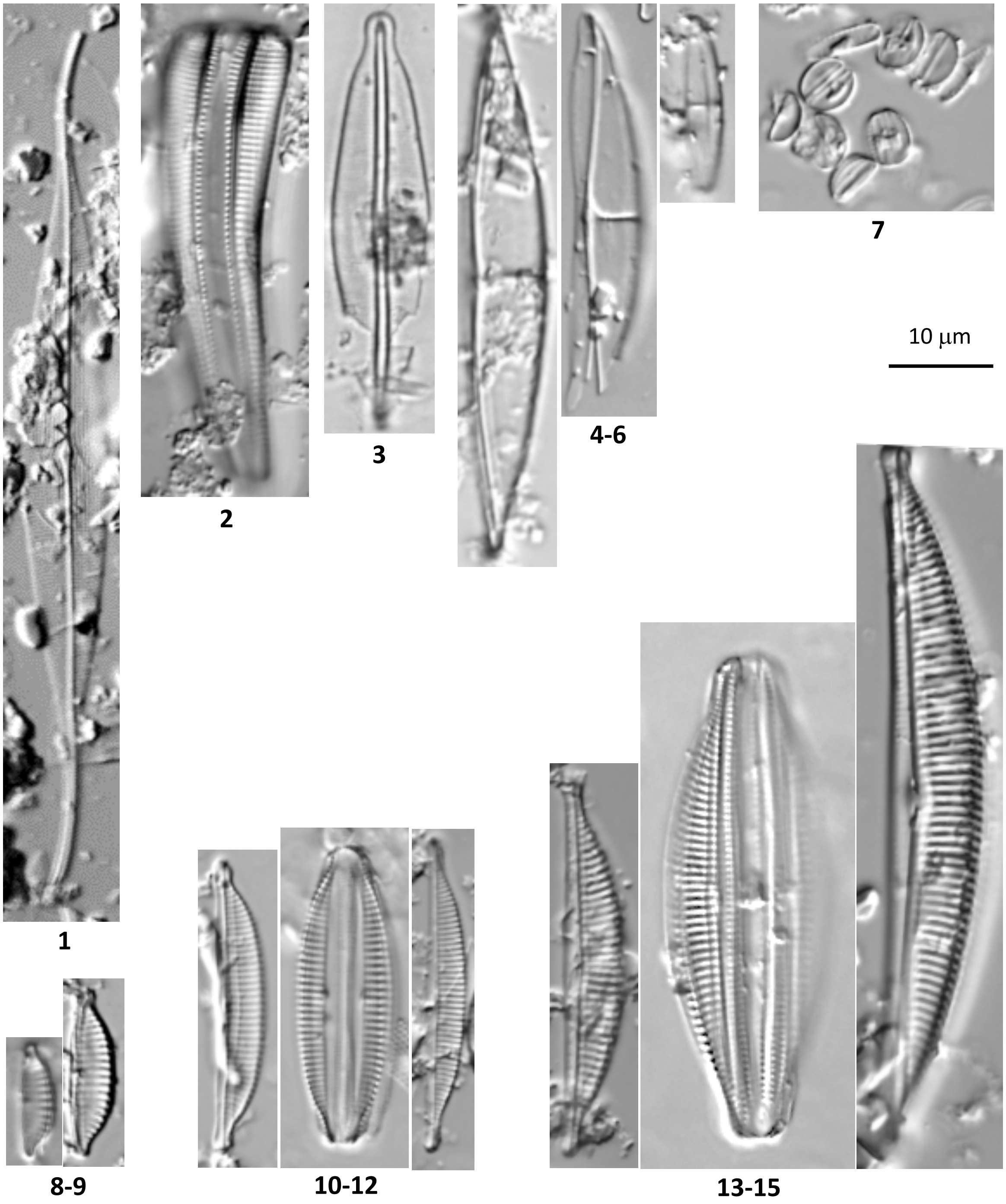

Fig.S9. 1: *Gyrosigma fasciola**. **2:** *Rhoicosphenia abbreviata*. **3:** *Frustulia saxonicai*. **4-6:** *Amphora staurophora**. **7:** *Amphora cf. exilitata**. **8-9:** *Amphora* cf. *wisei**. **10-12:** *Amphora* sp.58. **13-15:** *Amphora* cf. *pseudoholsatica*.

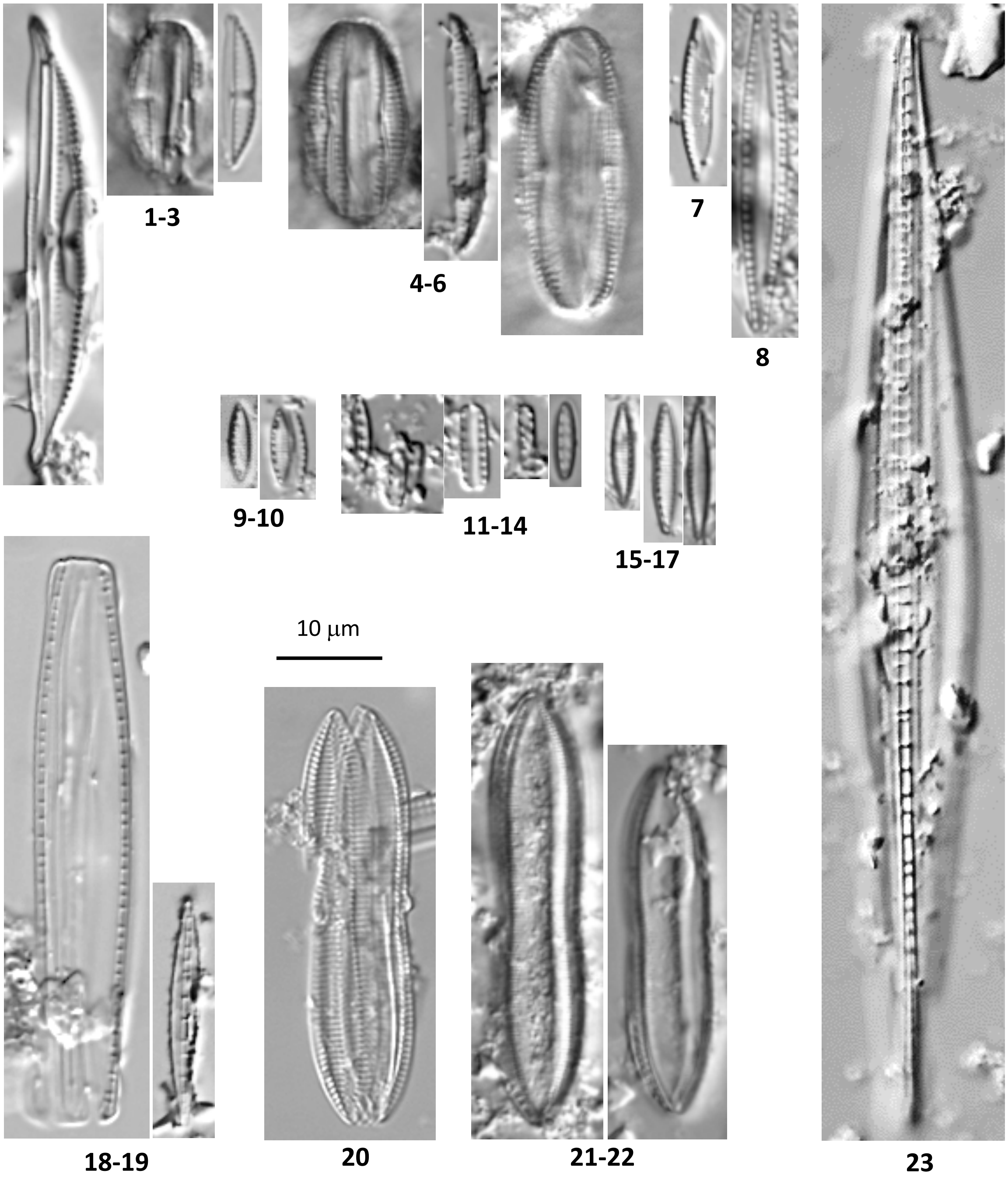

Fig.S10. 1-3: *Amphora maletracta* var. *constricta*. **4-6:** *Amphora inariensis*. **7-8:** *Nitzschia palea**. **9-10:** *Nitzschia frustulum**. **11-14:** *Nitzschia* cf. *nanodissipata**. **15-17:** *Nitzschia liebethrutii*. **18-19:** *Nitzschia dissipata**. **20:** *Tryblionella apiculata*. **21-22:** *Tryblionella marginulata* var. *subconstricta*. **23:** *Nitzschia angularis*.

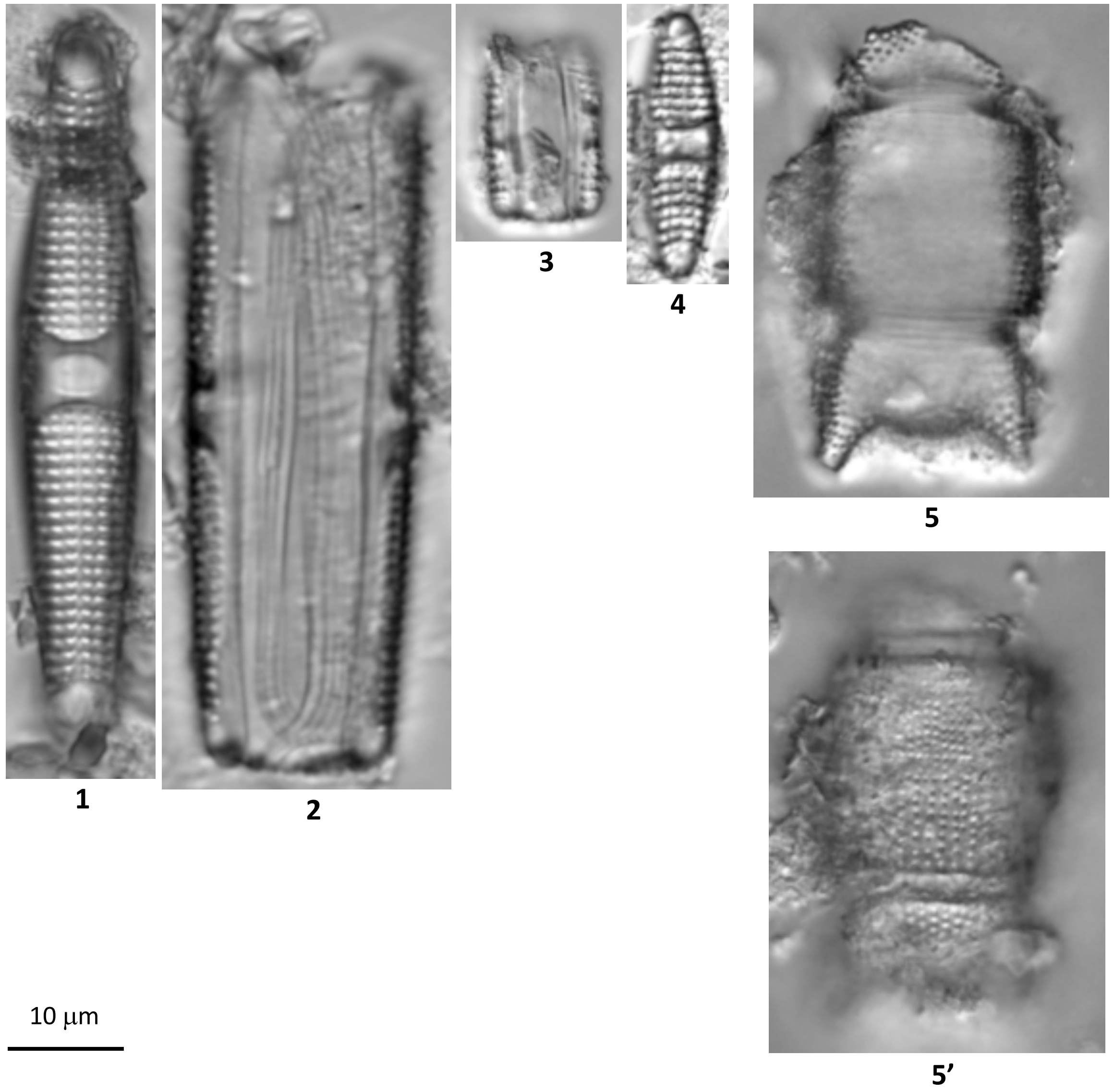

Fig.S11. 1-4: *Plagiogramma staurophorma*. **5-5’:** *Odontela aurita**.

Fig.S1-S11: Taxa with relative abundance >1%, taxa used for statistical analysis (relative abundance >2% in at least one site) are marked with an asterisk.

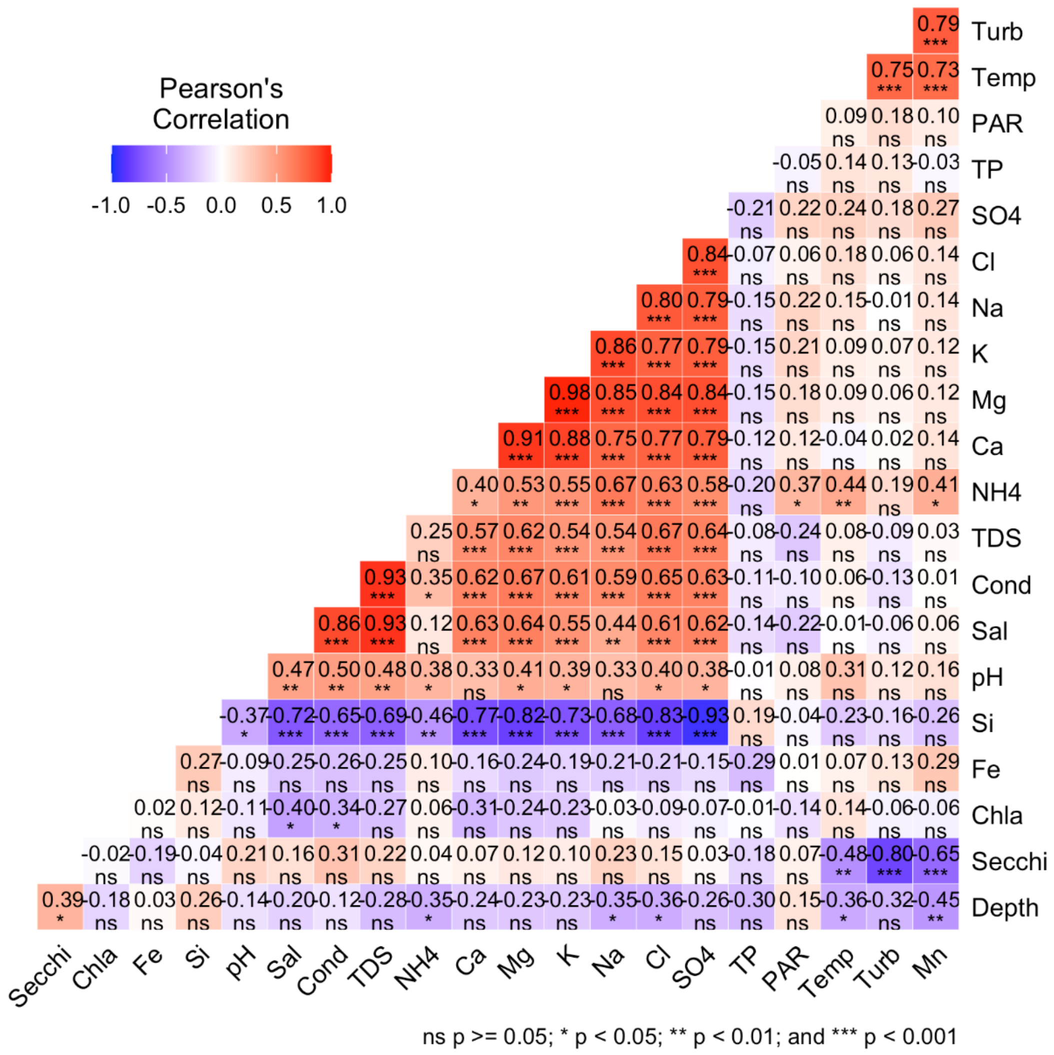

Fig.S12: Pearson correlation matrix of the 21 environmental variables for the 35 sites.

Table S1: The 55 benthic species used in the training set for the temperature model, their estimated optima and tolerances.

Species	Optima	tol_high	tol_down
Amicula vermicaluta	11.15031	11.59393	10.72366
Achnanthes* cf. *ricula	11.15633	11.51714	10.80682
***Achnanthes* sp.154**	11.44031	12.05981	10.85263
Achnanthes fimbriata	11.39495	11.69632	11.10134
Achnanthes* cf. *hauckiana	11.70471	12.48824	10.97034
Achnanthes lemmermannii	11.29524	11.96528	10.66273
Achnanthes minutissima	11.17633	11.92725	10.47269
Planothidium delicatulum	11.64218	12.30924	11.01127
***Amicula* sp.5**	11.30754	11.79765	10.83779
***Amicula / Fallacia* sp.4**	11.10659	11.70101	10.54236
Amicula specululum	11.0282	11.50662	10.56966
Amphora* cf. *exilitata	11.02452	11.61992	10.45962
Amphora* cf. *wisei	11.07368	11.56712	10.60129
Amphora staurophora	11.55708	11.94453	11.1822
Anaulus balticus	11.29712	11.77755	10.83629
Berkeleya rutilans	12.06691	12.68535	11.47861
***Cocconeis* sp.17**	11.34884	11.67757	11.02936
Cocconeis hauniensis	11.36144	12.19599	10.58399
Cocconeis* cf. *carminata	11.47136	11.84344	11.11097
***Cocconeis* sp.46**	10.86084	11.25	10.48513
Cocconeis costata	11.54098	12.27457	10.85125
Cocconeis euglypta	11.54248	12.23117	10.89256
Cocconeis peltoides	11.43163	12.03786	10.85593
Cocconeis scutellum	12.0404	12.80693	11.31975
Cocconeis scutellum* var. *parva	11.83067	12.63184	11.08032
Delphineis surirella	11.3836	12.11768	10.69398
***Fallacia* sp.06**	11.11059	11.46335	10.76868
Fallacia aequorea	11.56361	11.83092	11.30234
Fallacia escorialis	11.31792	11.80279	10.85298
***Fragilaria* sp.38**	11.98102	12.76452	11.24561
Fragilariforma virescens* var. *exigua 2	12.32676	12.7593	11.90889
***Girdle view Navicula* gp.19**	12.39382	12.6998	12.09522
***Girdle view Navicula* gp.2**	11.80816	12.42698	11.22016
***Achnanthes* sp.130**	11.17842	11.88263	10.51594
Gomphonemopsis exigua	12.11755	12.85139	11.42561
Grammatophora marina	12.19756	12.78883	11.63363
Grammatophora oceanica	12.29441	13.02042	11.60888

Gyrosigma fasciola	12.22131	12.58503	11.86811
***Navicula* sp.491**	10.86271	11.08018	10.64951
Navicula* cf. *flanatica	11.75848	12.27599	11.26279
Navicula* cf. *transitans* var. *deresa	11.9832	12.3793	11.59977
Navicula directa	11.86157	12.65792	11.11532
Navicula germanopolinica	12.05085	12.67402	11.45831
Navicula perminuta	11.49117	12.30306	10.73286
Nitzschia dissipata	11.75287	12.20282	11.31951
Nitzschia frustulum	11.93385	12.64201	11.26535
Nitzschia nanodissipata	10.77999	11.2479	10.33155
Nitzschia palea	11.91559	12.67598	11.20081
Odontella aurita	12.25732	12.76128	11.77327
Parlibellus delognei	11.63077	12.23538	11.05603
Pinnularia quadratarea	11.60943	12.35465	10.90917
Pseudostaurosira* cf. *brevistriata	12.10169	12.41312	11.79808
Tabellaria floculosa	11.72909	12.47248	11.03
Tabularia fasciculata	12.348	12.97853	11.7481
Tabularia waernii	11.78216	12.66104	10.96429

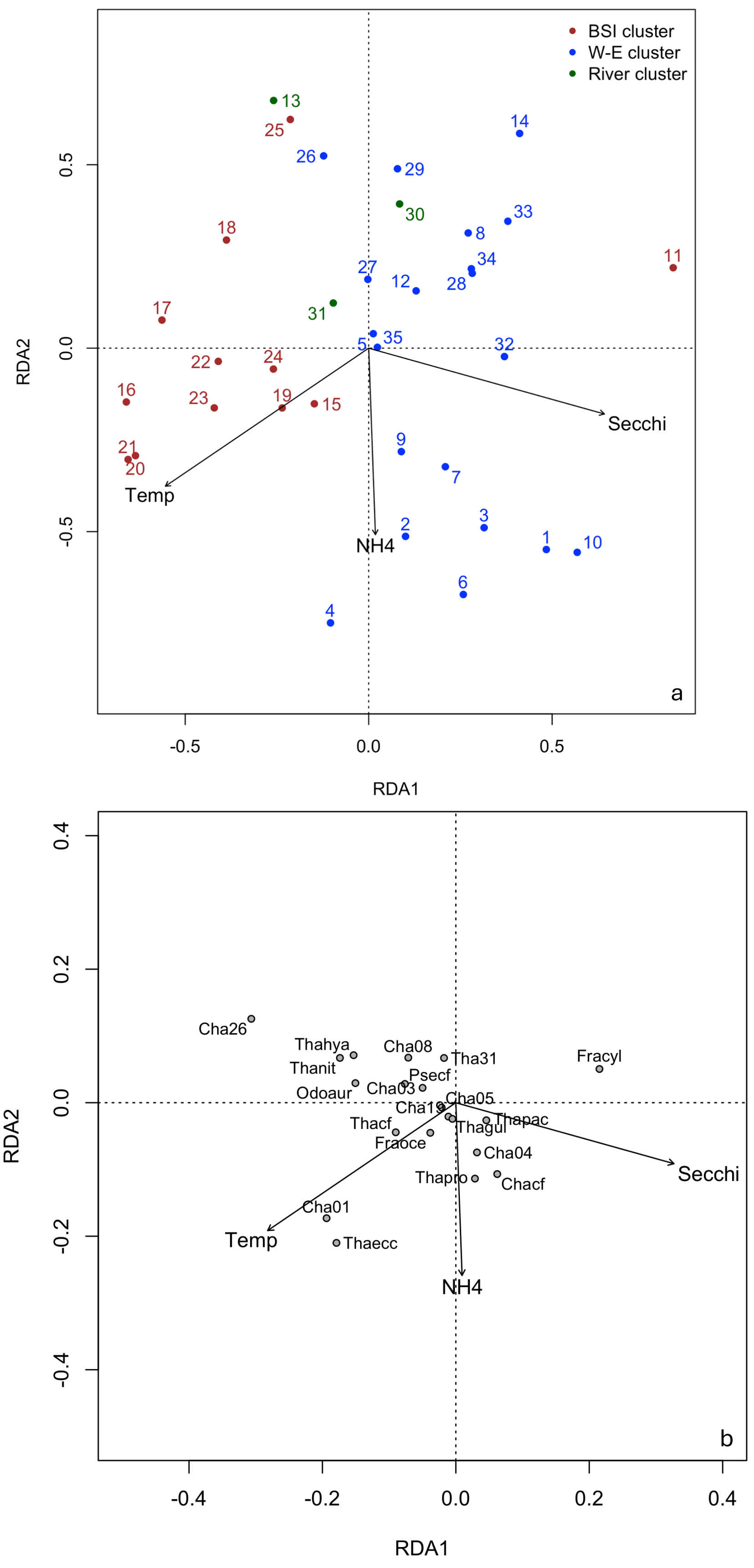
BSI cluster
W-E cluster
River cluster
RDA1
RDA2
Temp
NH4
Secchi
a
b
Cha26
Thahya
Thanit
Odoaur
Cha08
Tha31
Psecf
Cha03
Cha05
Cha19
Thacf
Fraoce
Thagul
Thapac
Cha04
Thapro
Chacf
Cha01
Thaecc
Fracyl

Fig.S13: Redundancy analysis (RDA) showing the relationships between sites (a) and planktonic species (b) between the three selected environmental variables.
(Chae26 = *Chaetoceros* sp.26; Thahya = *Thalassiosira hyalina*; Thanit = *Thalassionema nitzschioides*; Odoaur = *Odontella aurita*; Cha08 = *Chaetoceros* sp.08; *Chae03* = *Chaetoceros* sp.03; *Cha05* = *Chaetoceros* sp.05; *Cha19* = *Chaetoceros* sp.19; Psecf = *Pseudostaurosira* cf. *brevistriata*; Tha31 = *Thalassiosira* sp.31; Fracyl = *Fragilariopsis cylindrus*; Thapac = *Thalassiosira pacifica*; Thagui = *Thalassiosira guillardii*; Cha04 = *Chaetoceros* sp.04; Chacf = *Chaetoceros* cf. *furcellatus*; Thapro = *Thalassiosira proschkinae*; Thaecc = *Thalassiosira eccentrica*; Cha01 = *Chaetoceros* sp.01; Fraoce = *Fragilariopsis oceanica*; Thacf = *Thalassiosira* cf. *hyperborea var. pelagica*)

Table S2: Variance explained for each variable used in the RDA, their significance and the eigenvalues ratio test for developing transfer functions.

**Variable**	**Variance explained (%)**	**RDA1 (%)**	**PC1**	λ_1 / λ_2	***p value***
Temperature	8.12	1.93	5.95	0.33	0.01
Secchi	8.81	2.10	5.69	0.37	0.01
Nh_4^+	5.95	1.42	6.10	0.24	0.05
